# Advancing common bean (*Phaseolus vulgaris* L.) disease detection with YOLO driven deep learning to enhance agricultural AI

**DOI:** 10.1038/s41598-024-66281-w

**Published:** 2024-07-06

**Authors:** Daniela Gomez, Michael Gomez Selvaraj, Jorge Casas, Kavino Mathiyazhagan, Michael Rodriguez, Teshale Assefa, Anna Mlaki, Goodluck Nyakunga, Fred Kato, Clare Mukankusi, Ellena Girma, Gloria Mosquera, Victoria Arredondo, Ernesto Espitia

**Affiliations:** 1 International Center for Tropical Agriculture, Km 17 Recta Cali-Palmira, Cali, Colombia; 2 International Center for Tropical Agriculture, Arusha, Tanzania; 3 Department of Horticulture, Agricultural College and Research Institute, Tamil Nadu Agriculture University, Vazhavachanur, Tiruvannamalai, Tamil Nadu India; 4 International Center for Tropical Agriculture, Kawanda, Uganda

**Keywords:** Computational neuroscience, Data processing, Image processing, Machine learning, Biological techniques, Biotechnology, Plant sciences, Plant breeding

## Abstract

Common beans (CB), a vital source for high protein content, plays a crucial role in ensuring both nutrition and economic stability in diverse communities, particularly in Africa and Latin America. However, CB cultivation poses a significant threat to diseases that can drastically reduce yield and quality. Detecting these diseases solely based on visual symptoms is challenging, due to the variability across different pathogens and similar symptoms caused by distinct pathogens, further complicating the detection process. Traditional methods relying solely on farmers’ ability to detect diseases is inadequate, and while engaging expert pathologists and advanced laboratories is necessary, it can also be resource intensive. To address this challenge, we present a AI-driven system for rapid and cost-effective CB disease detection, leveraging state-of-the-art deep learning and object detection technologies. We utilized an extensive image dataset collected from disease hotspots in Africa and Colombia, focusing on five major diseases: Angular Leaf Spot (ALS), Common Bacterial Blight (CBB), Common Bean Mosaic Virus (CBMV), Bean Rust, and Anthracnose, covering both leaf and pod samples in real-field settings. However, pod images are only available for Angular Leaf Spot disease. The study employed data augmentation techniques and annotation at both whole and micro levels for comprehensive analysis. To train the model, we utilized three advanced YOLO architectures: YOLOv7, YOLOv8, and YOLO-NAS. Particularly for whole leaf annotations, the YOLO-NAS model achieves the highest mAP value of up to 97.9% and a recall of 98.8%, indicating superior detection accuracy. In contrast, for whole pod disease detection, YOLOv7 and YOLOv8 outperformed YOLO-NAS, with mAP values exceeding 95% and 93% recall. However, micro annotation consistently yields lower performance than whole annotation across all disease classes and plant parts, as examined by all YOLO models, highlighting an unexpected discrepancy in detection accuracy. Furthermore, we successfully deployed YOLO-NAS annotation models into an Android app, validating their effectiveness on unseen data from disease hotspots with high classification accuracy (90%). This accomplishment showcases the integration of deep learning into our production pipeline, a process known as DLOps. This innovative approach significantly reduces diagnosis time, enabling farmers to take prompt management interventions. The potential benefits extend beyond rapid diagnosis serving as an early warning system to enhance common bean productivity and quality.

## Introduction

The common bean (*Phaseolus vulgaris* L.) serves as one of the essential staple foods in Africa, particularly in rural and urban Eastern African and Latin American communities, providing vital dietary protein for over 300 million individuals^1–4^. Regardless of its significance, common bean crops are vulnerable to various diseases, resulting in significant losses in yield and quality. Among the devastating diseases affecting common bean production, common bacterial blight (CBB) and angular leaf spot (ALS) pose significant threats in Ethiopia^5,6^. CBB has been documented as a major seed-borne disease, causing yield losses ranging from 30 to 70% in susceptible cultivars globally^7^. Although ALS causes infrequent damage in the USA and Europe, it leads to significant yield losses of 50% to 60% in Africa^8^.

The symptoms of common bean diseases vary across different pathogens, presenting a significant challenge in pinpointing their specific causes. In addition, similar symptoms induced by various pathogens also make it challenging for farmers and researchers to precisely identify the underlying disease. Moreover, environmental factors such as temperature, humidity, and soil conditions complicate disease diagnosis, sometimes mimicking those caused by pathogens. The influence of environmental factors such as temperature, humidity, and soil conditions, the similarity of symptoms caused by abiotic factors like nutrient deficiency, and the difficulty in distinguishing lesions produced by bacteria and fungi, along with limited accessibility to advanced diagnostic surveillance tools and expertise, aggravate the complexity in diagnosing these diseases properly^9^. Without timely and precise disease identification, these complexities exacerbate the disease, leading farmers to struggle with their crop health.

Therefore, early, and precise disease identification is crucial for mitigating the disease spread, safeguard the agricultural productivity, and minimizing their negative impact on farmers’ livelihoods, ensuring food security^10–13^. The need for such advancements necessitates the development of a low-cost automatic system capable of skillfully identifying and classifying diseases early, designed for easy operation by farmers, and leveraging the widespread availability of smartphones and internet accessibility. An imperative task lies in the integration into mobile apps, enabling the provision of near-expert supervision in real field conditions, especially across vast areas and during the early stages of crop growth^14–19^. These algorithms offer timely guidance to farmers after analyzing specific diseases, suggesting essential disease mitigation strategies or adjustments to cultural practices to enhance crop yield. The combination of AI technology and mobile applications capitalizes on the widespread availability of smartphones and internet connectivity, ensuring accessibility and usability for farmers^20,21^.

One such technique is the automatic detection and identification of plant diseases using deep learning models and image processing methods^22^. In recent times, AI-based deep learning models have revolutionized plant disease diagnosis, as researchers are increasingly using deep learning models to identify and categorize plant diseases in major crops^23,24^, including Rice^25–27^, Wheat^28,29^, Maize^30^, Tomato^31–34^, Banana^16,35^, Apple^34,35^, Grapes^15,36^, Citrus^37–39^, Mango^40,41^, Tea^42–44^, Cucumber^45,46^, Cassava^47,48^, Ginger^18,47^, Sugarcane^48,49^, Papaya^50^, and Pearl Millet^51^. Despite their effectiveness in diagnosing and classifying various crop diseases, these techniques still encounter limitations in detecting and recognizing common bean diseases in real-field environments. This is due to several factors, including complex backgrounds, co-occurring diseases on individual leaves, dense foliage, and large-scale variations.

Therefore, overcoming the limitations of current AI-based recognition systems for crop diseases, particularly in the case of common bean, requires a transition towards curated image datasets specifically tailored for real-field environments. As a result, most disease detection models currently deployed in agricultural settings often lack the necessary adaptability to handle the diverse situations encountered by farmers on a daily basis^35^. These limitations arise from the models being designed for specific crop varieties or optimized for operation in controlled, uniform environments.

To address this challenge and construct robust disease detection models, the critical step lies in the acquisition of a comprehensive and diverse dataset. This dataset should encompass a broad spectrum of image data, capturing healthy and diseased plant specimens from a wide range of crop varieties. Crucially, image capture should occur under a variety of environmental conditions, with particular emphasis on conditions typically associated with disease outbreaks, ensuring a holistic representation of potential disease indicators.

While the collection of a diverse image dataset is essential, it represents only one facet of the challenge. To ensure the accuracy and reliability of the training data, meticulously curated image pre-screening by expert plant pathologists is indispensable. This rigorous approach to data preparation directly translates to the effectiveness and adaptability of the resulting disease detection models across different agricultural scenarios^35^. Recent advancements in deep learning models offer promising solutions to overcome the challenges faced by disease detection models in real-world agriculture. One particularly promising approach lies in the development of efficient one-stage deep learning (DL) networks. Traditionally, deep learning-based image detection networks have been categorized into two main types: two-stage and one-stage networks. Two-stage category, such as the well-established Faster R-CNN, initially, prioritize high accuracy by first pinpointing potential region of interest (disease locations) on the plant. While this approach delivers superior accuracy, it comes at the cost of increased computational demands^52–54^. In contrast to two stage models, one-stage models such as YOLO (You Only Look Once) and SSD (Single Shot Detectors) represent the forefront of advancements in object detection. These models achieve their superior efficiency by integrating the process of identifying potential disease locations and classifying these locations more efficiently within a single streamlined work. This focus on efficiency makes YOLO series particularly well-suited for challenging environments, enabling real-time disease detection, such as leaf diseases^35,44, 55–59^.

Within the YOLO family, YOLO-NAS stands out as a recent innovation, distinguished by its advanced features for automated neural architecture optimization. Although YOLO-NAS has only recently been applied in agricultural contexts, as evidenced by its role in advancing disease identification in fava bean crops^60^, its incorporation into agricultural technology represents a significant breakthrough, greatly enhancing the potential for improved disease detection and management in this sector^44^. The model’s ability to achieve a balance between speed and accuracy is crucial for real-time disease monitoring in the field, allowing for timely interventions and crop management decisions^35,44, 52–59^. Therefore, in this study, we aim to recognize the promising potential of YOLO-NAS deep-learning architecture for achieving a favorable trade-off between inference latency and classification precision in real-time agriculture disease detection settings. By harnessing the power of AI for disease detection in common bean crops, this research seeks to contribute to the development of effective and practical solutions for managing these diseases in key regions like Latin America and Africa. We will achieve this by focusing on the following objectives:

1. Compile a comprehensive dataset enriched with expert annotations, encompassing a wide range of common bean leaf and pod diseases prevalent in high-risk regions, particularly in Africa and Colombia.
2. Develop and rigorously evaluate advanced YOLO-based object detection models (YOLOv7, YOLOv8, and YOLO-NAS) customized for accurately identifying common bean diseases in varied and demanding real-farm settings.
3. Employ robust evaluation metrics to determine the most effective framework for high-accuracy disease detection, informing future research and practical applications in crop disease detection.
4. Furthermore, the promising YOLO models will be integrated into an Android AI application for deployment and validation of real-world field detection capabilities, showcasing the complete pipeline of Deep Learning Operations (DLOPs).

## Materials and methods

### System description of deep transfer learning approach

**Figure 1:**
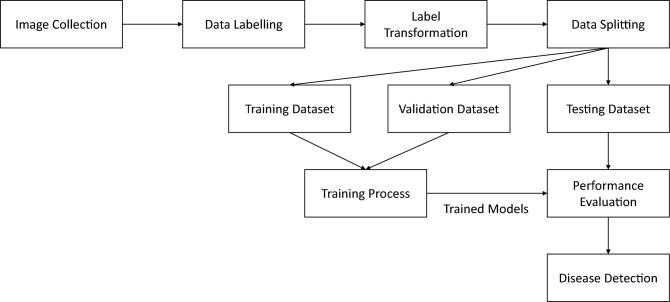
Overview of training, testing, and validation processes for proposed YOLO models.

Our Deep Transfer Learning (DTL) system features five major common bean diseases, along with their respective healthy counterparts, as outlined in Table 1. Recognizing that certain diseases, such as Angular Leaf Spot (ALS), can affect both the leaves and pods of bean plants, we devised separate models to address these variations. As a result, the dataset comprises six distinct classes for leaves and two for pods, as elaborated in Table 1. For a visual representation of our DTL system’s architecture and workflow, refer to Fig. 1. This system architecture is specifically designed to effectively process and analyze the complex variations present in our dataset, ensuring accurate disease detection and classification for both leaves and pods of common bean crops.

**Table 1:** Description of annotated common bean datasets used in this study.

Model/classes	Images collected		Whole annotations		Total whole annotations	Micro annotations		Total micro annotations
	Leaf	Pod	Leaf	Pod		Leaf	Pod	
Healthy	986	1150	2030	815	2845	2030	815	2845
ALS	1302	1021	1502	1241	2743	13,689	2970	16,659
CBB	1430		2667		2667	2395		2395
CBA	1287		431		431	4677		4677
CBMV	1300		691		691	691		691
BR	1088		592		592	6786		6786
Total	**7393**	**2171**	7913	2056	**9969**	30,268	3785	**34,053**

Significant values are in bold.

### Dataset collection for common bean disease detection

**Figure 2:**
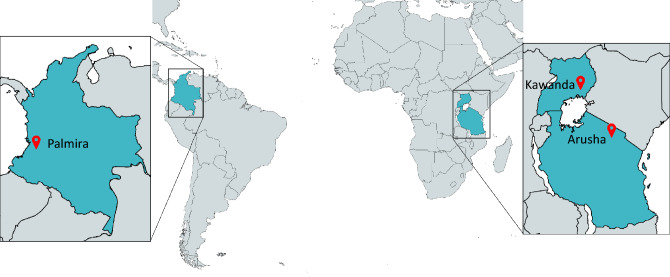
Geographic representation of image collection and model testing sites.

The construction of a robust model for common bean disease detection involved compiling a comprehensive image dataset from three key locations: Palmira (Colombia), Arusha (Tanzania), and Kawanda (Uganda), as illustrated in Fig. 2. This dataset, which consisted of approximately 9,500 real-field images, was collected by trained field technicians and validated by pathology experts associated with the Alliance Bioversity International and CIAT, as outlined in Table 1.

#### Image sources and environmental diversity

The images were sourced from diverse field settings, including research stations and farmers’ fields, presenting a wide array of environmental conditions. To ensure a diverse range of images, our dataset includes pictures captured using various mobile phones, resulting in different resolutions (see Supplementary Table 1). Additionally, the images exhibit a wide spectrum of lighting conditions, influenced by factors such as the time of day, seasonal factors like temperature and humidity, and the unique geographical aspects of the locations in Africa and Colombia.

**Figure 3:**
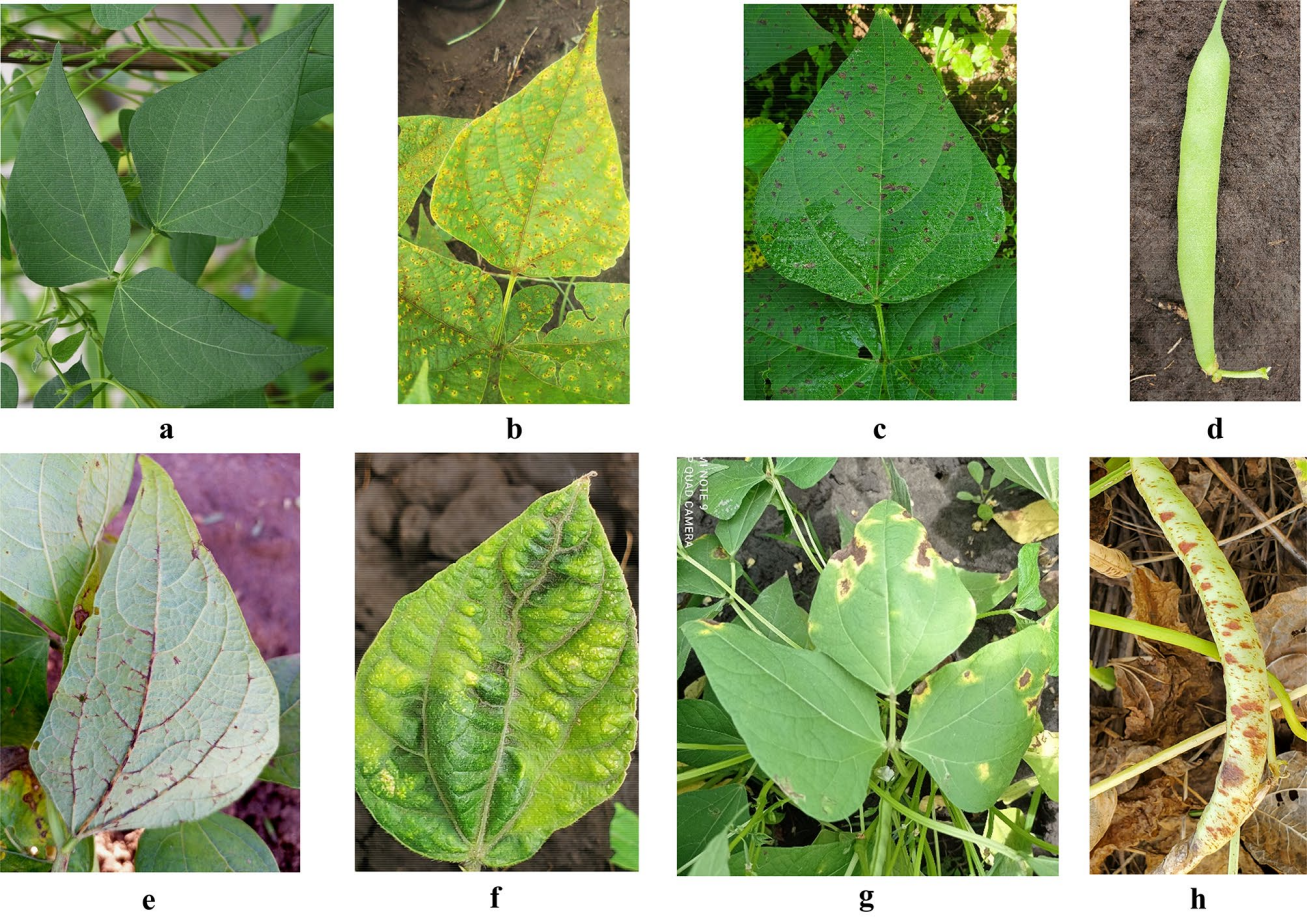
Real field images of common bean disease. (**a**) Healthy leaf, (**b**) common bean rust, (**c**) angular leaf spot (ALS), (**d**) healthy pod, (**e**) common bean anthracnose (CBA), (**f**) common bean mosaic virus (CBMV), (**g**) common bean bacterial blight (CBB), (**h**) angular leaf spot (ALS) in pod.

An example of the images belonging to the dataset, along with additional details for each disease, can be seen in Fig. 3 and in Supplementary Table 2, respectively.

The dataset encompasses images from various bean varieties, spanning various plant growth stages and a spectrum of environments in Africa and Colombia. Supplementary Table 2 provides a comprehensive overview of the included variations, ensuring the dataset captures the multifaceted conditions conducive to these diseases. This compiled dataset serves as a crucial resource for developing and validating our AI model, designed to identify and manage common bean diseases under real-world conditions.

### Dataset annotation

#### Whole annotations

**Figure 4:**
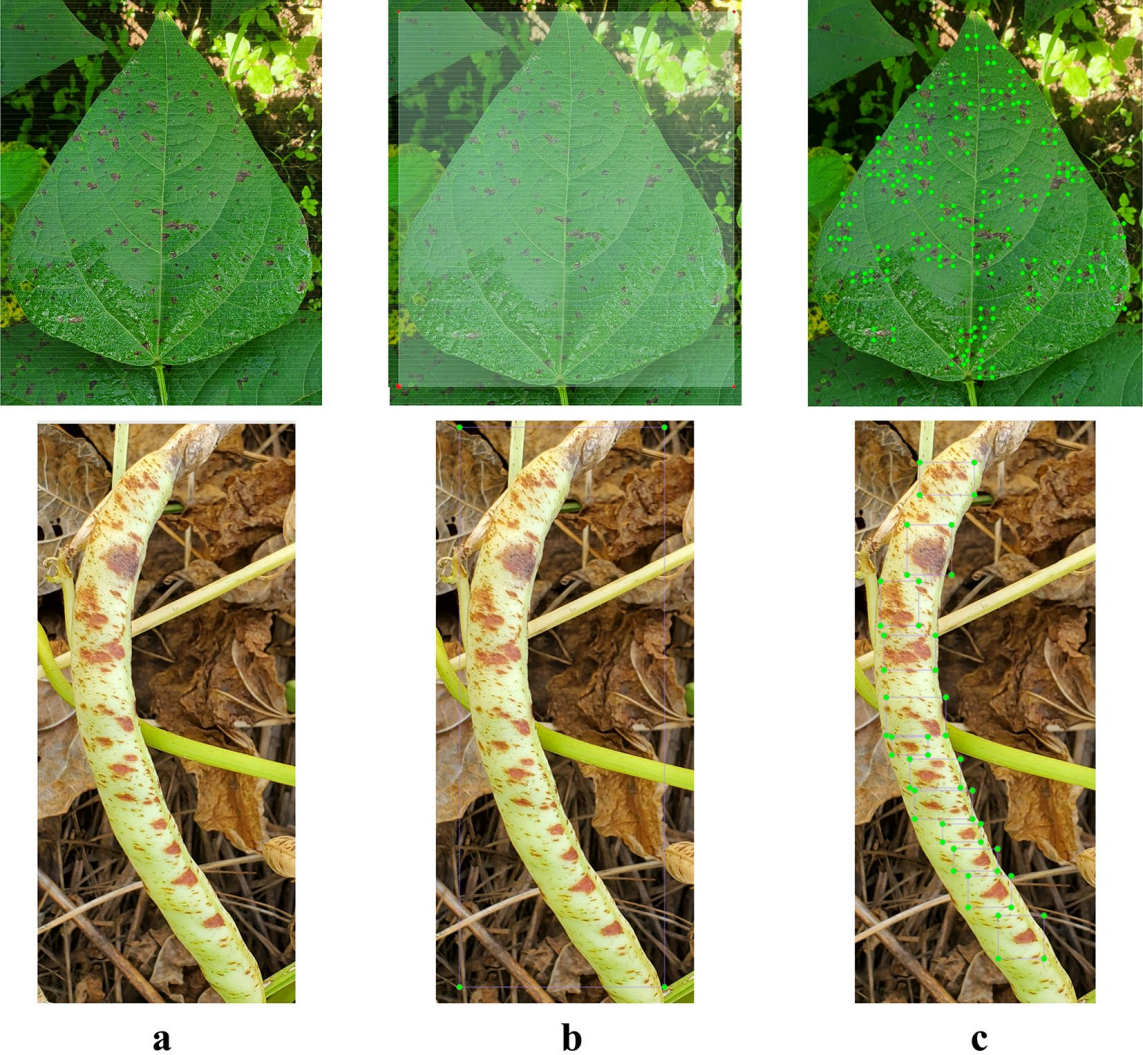
Demonstration of the annotation process as ALS class (leaf and pod) examples. (**a**) Original raw image; (**b**) whole class annotation; (**c**) micro or focused annotations.

The annotation process for disease or infection in our images involved using the *‘Labellmg’* software. This tool enabled us to create bounding boxes around affected areas within the entire training set. Each image (Fig. 4a) underwent detailed manual annotation, which included drawing bounding boxes and assigning corresponding class labels (Fig. 4b). The resulting annotations were saved as XML files and then converted to YOLO format to ensure compatibility with the models.

The labeling process focused on precision, aiming to encapsulate common bean leaves and pods within the smallest surrounding rectangle to minimize background inclusion. This approach resulted in approximately 9969 annotations in total (Table 1).

#### Micro annotations

In our study, we propose that micro-annotations improve the precision of detecting leaf spot diseases in common beans. Because these diseases are caused by diverse pathogens, which exhibit complex patterns and localized lesions, a detailed analysis is essential for accurate identification. To perform micro annotations, we marked and labeled individual spots, lesions, or discolorations on the leaf surface (Fig. 4c), providing detection models with deeper insights into disease characteristics and enhancing classification accuracy. Approximately 34,053 micro annotations were thoroughly created through extensive annotation efforts (see Table 1).

### Data augmentation

In our study, we employed data augmentation to achieve a balanced representation of healthy and diseased classes, utilizing the *imageaug* Python library tailored for machine learning experiments. This library offers a versatile range of augmentation techniques that can be seamlessly integrated and executed either randomly or sequentially^57^. Various techniques were applied, including flipping images over vertical and horizontal axes to create a mirror effect, as well as adjusting pixel brightness through multiplication by random numbers to introduce variations in darkness or lightness across images.

### YOLO architectures

In this study, we focused on training models for classifying leaf and pod diseases using three different CNN architectures: YOLOv7, YOLOv8, and YOLO-NAS. These models were applied to four distinct datasets: whole-annotated leaf images, whole-annotated pod images, micro-annotated leaf images, and micro-annotated pod images (Table 1).

For the training process, we utilized PyTorch, taking advantage of its dynamic computational graph and compatibility with NVIDIA CUDA for accelerated GPU processing. We started with pre-trained models initially developed on the COCO dataset and employed a transfer learning approach to adapt these models to our specific datasets. Throughout the training process, we adjusted specific configurations to optimize each model.

For the leaf datasets, we used a batch size of 8, whereas for the pod datasets, we used a batch size of 1. This decision was made due to the smaller number of pod images and the complexity of the problem. To ensure thorough training, we employed 100 epochs for the YOLOv7 and YOLOv8 models on leaf datasets, and 70 epochs for YOLO-NAS. For the more complex pod datasets, we extended training to 300 epochs. This uniform training approach was applied across all three YOLO models for each dataset type.

### Model training

Splitting data in machine learning poses challenges in avoiding overfitting, underfitting, or generalization issues. However, various sophisticated statistical sampling methods can help address these concerns. In the development of our CB disease detection model, we divided our dataset into training (Ttr), validation (Tv), and testing (Tt) sets in proportions of 70%, 20%, and 10%, respectively. To ensure simplicity and efficiency in implementation, we opted for randomly selecting the images belonging to each of the data sets.

### Performance metrics

#### Loss function

In this study, we assess various loss functions aimed at quantifying the disparity between predicted outputs and ground truth values, each serving to evaluate different aspects of model performance. These include the Classification Loss, which evaluates errors in classifying objects into categories; the IoU Loss, which measures the overlap between predicted and ground truth bounding boxes; the Dual Focal Loss, designed to address class imbalance by prioritizing hard negatives during training, thereby enhancing classification accuracy for unbalanced classes; the Objectness Loss, quantifying errors in predicting the “objectness” score of a bounding box; and the Box Loss, which assesses errors in localizing bounding boxes. For YOLO-NAS, the total loss combines classification, dual focal, and IoU losses.

#### mAP score

The mean average precision (mAP) serves as a key validation metric for assessing the efficacy of object detection models, particularly in the context of detecting common bean diseases. This metric evaluates the overall performance of the model across all classes, providing insights into its ability to accurately detect and recall objects from various categories. A high mAP score indicates that the model demonstrates proficiency in detecting objects across all classes with precision and recall. The mAP is calculated as the mean of average precisions (APs) computed for each individual object class.

#### Precision and recall

Precision measures the accuracy of the positive predictions made by the model. Recall measures the fraction of actual positive instances that were correctly identified by the model, relative to all positive examples. The equations for mAP, precision and recall are shown for a better understanding:

$$Precision=\frac{True\, Positives (TP)}{True \,Positives \left(TP\right)+ False\, Positives (FP)},$$

$$Recall=\frac{True \,Positives (TP)}{True\, Positives (TP) + False\, Negatives (FN),},$$

$$mAP= \frac{1}{\#classes} \sum_{K=1}^{K=\#classes}{AP}_{K}.$$

#### Confidence score

In object detection tasks, the confidence score, also referred to as the prediction probability, serves as a crucial indicator of the model’s certainty regarding the presence and classification of objects within bounding boxes in an image. This numerical value, ranging from 0 to 1, conveys the level of confidence of the model predictions. A score of 0 indicates no confidence in the prediction, while a score of 1 indicates absolute certainty. Metrics such as precision and recall scores are dependent on the choice of an appropriate confidence threshold.

#### Confusion matrix

In addition to the mean Average Precision (mAP) score, we calculated confusion matrices (CMs) to further evaluate the performance of each assessed model in object detection tasks. The CM generation protocol involved the following steps.

Detections were matched with ground-truth boxes based on class alignment and an Intersection over Union (IoU) score threshold of ≥ 0.5. Any detections failing to meet this threshold below this were discarded. Each ground-truth box was paired with the detected box exhibiting the highest IoU score in cases of multiple matches, ensuring elimination of any redundant pairings. The CM was updated to accurately represent these pairings, categorizing a detection as correct if its IoU with a ground-truth box was ≥ 0.5. Subsequently, the CM was normalized to facilitate fair comparison and comprehensive analysis across models and datasets.

### Deployment and testing of the YOLO-NAS model in the AI app

In a significant step towards practical implementation in real-world agricultural settings, we have developed the Beta version of the CB TUMAINI AI app for Android-based smartphones. This app is designed to accurately identify and classify common bean diseases in the field. Once validated, this CB model from this app can seamlessly integrate into our already-released AI app (https://play.google.com/store/apps/details?id=ciat.cgiar.org.tumaini&hl=es_PE).

Our AI application development process begins with user interface (UI) design, focusing on creating a smooth and intuitive user-friendly experience utilizing the dynamic UI framework provided by Flutter. This allows us to create a cross-platform interface, ensuring the app functions seamlessly on operation across various android devices without compromising user experience. Following the UI design, we integrate AI features into the application. This includes utilizing the YOLO-NAS model for accurate disease classification of common bean crops directly within the app. All model processing for disease detection was carried out in a cloud server located in Vietnam. This decision considered the proximity to the regions of interest, primarily Africa and Asia, where these diseases are most prevalent, and the limitations of the YOLO-NAS model, which currently cannot run offline directly on smartphones. This server is part of a commercial cluster dedicated to selling cloud services for applications that require high processing power.

#### Model efficacy

To assess the effectiveness of our YOLO-NAS model for common bean disease detection, we conducted extensive rigorous testing using a unique dataset obtained from already known disease hotspots in Africa and Asia, ensuring the model’s adaptability to real-world scenarios. This dataset comprised of 300 images for the whole leaf model and 100 for the whole pod model, covering all classes of interest from disease hotspots. We optimized real-time data processing for enhanced mobile app performance. By implementing techniques such as caching and prefetching, we significantly reduced load times and improved the app’s responsiveness, leading to a better user experience. Additionally, we collected feedback through user testing, which played a crucial role in further refining the app’s usability and functionality.

### Software and hardware systems used in this study

The primary programming language for algorithm implementation and data wrangling scripts was Python 3.9. For the re-training of models, we utilized PyTorch, a robust deep learning framework developed by Facebook. PyTorch offers extensive support for both CPU and GPU training and inference, enhancing the efficiency and scalability of our experiments. Detailed insights into PyTorch can be found in Ref.^61^. In addition, simple implementations of the YOLO-NAS models were utilized for this study. The YOLO-NAS model, as referenced in Ref.^62^, was implemented using the SuperGradients library, developed by Deci-AI^63^. Additionally, the YOLOv8 model, as detailed in Ref.^64^, was implemented using the Ultralytics package^65^. Similarly, the YOLOv7 model, outlined by Ref.^66^, was implemented using the original repository of WongKinYiu. The list of hardware and software used in this study are depicted in Table 2.

**Table 2:** Hardware and software utilized in this study.

DL modeling hardware	
RAM memory	100 GB
Processor	Intel Xeon E5-2667 v4 @ 3.20 GHz × 16
GPU	NVIDIA Tesla M60
DL modeling software	
OS	Windows Server 2019 https://www.microsoft.com/es-es/evalcenter/download-windows-server-2019
Programming language	Python v3.9.17 https://www.python.org/downloads/release/python-3917/
Labelling software	LabelImg v1.8.6 https://github.com/HumanSignal/labelImgLabelImg v1.8.6 https://github.com/HumanSignal/labelImg
Deep learning libraries	PyTorch v1.12.1 with CUDA 11.6 https://pytorch.org/get-started/previous-versions/ SuperGradients v3.1.3 https://docs.deci.ai/super-gradients/latest/index.html#quick-installation Ultralytics v8.1.27 https://pypi.org/project/ultralytics/8.1.27/

### Research involving plant statement

The authors affirm that all requisite permissions and licenses for the collection of plants, pod specimens, and their accompanying images, utilized in this study, have been duly obtained in adherence to relevant regulations and guidelines. Additionally, the authors confirm that the species utilized in this study are not endangered.

## Results and discussion

### Building a diverse dataset for common bean disease detection

Common beans, also known as nearly perfect food for their nutritional richness, stand as a linchpin for economic stability, uplifting the livelihoods of smallholder farmers worldwide^67^. Yet, the specter of disease looms large over common bean cultivation, presenting a daunting challenge. Detecting and validating these diseases constitutes a second major hurdle for pathologists, a complex and time-consuming endeavor that invariably demands expert supervision.

To expedite disease detection and facilitate timely management interventions, a comprehensive image dataset, recognizing the inadequacy of existing public resources like the PlantVillage dataset for common bean diseases, collaborating with CGIAR bean network experts were developed. Collectively, 9564 original field images from diverse disease hotspots were amassed. A subset of these images formed our annotated image dataset, outlined in Table 1. These images were curated by expert pathologists for precise disease identification.

To ensure heterogeneity, images were captured in real-field settings, documenting the intricacies of actual field conditions and plant interactions across different growth stages (Supplementary Table 2). Additionally, various cameras were used to capture the images, introducing variations in image quality and background complexity. A realistic spectrum of disease presentations within the context of agricultural variability depicts the challenges that crops encounter in the dynamic environmental conditions during various growth stages, which is an essential step in developing a globally beneficial, mobile-assisted disease detection tool^35^. This strategic preparation equips our model for deployment in diverse and unpredictable environments where common beans are cultivated.

### Micro-annotation and augmentation techniques for enhancing CNN performance

To enhance the performance of our CNN in identifying common bean diseases, we implemented micro-annotations and data augmentation techniques to create a more robust training dataset. Data augmentation techniques, such as flipping and brightness adjustments, were applied strategically to diversify the dataset and address overfitting to effectively generate additional data variations (Table 1). These techniques were selectively applied to datasets with least amount of data to maximize their impact. This includes CBMV, Rust, and ANTH classes for whole leaf annotations; Rust class for micro leaf annotations; and healthy class for pod annotations. These augmentation strategies enriched the training dataset, introducing diversity into the samples and enhancing the performance and generalization of the deep-learning models used for disease detection.

Conversely, micro annotations focus on identifying specific disease symptoms at a micro level, which are essential for training highly accurate and sensitive models. While manually annotating each small symptom can be challenging due to resource constraints, micro annotations have the potential to enhance the generalization of models, allowing them to recognize a wider range of disease variations. However, their performance is highly dependent on the factors like data complexity, data quantity and annotation quality.

The dataset was split (70% training, 20% testing, and 10% validation) to ensure representation across different disease classes. Each image underwent rigorously validation by a bean phytopathologist, resulting in a comprehensive set of 44,022 annotations before data augmentation, and expanding to 54,264 after data augmentation (Table 1). This labor-intensive annotation process, conducted by three experts over 4 months, underpins the dataset’s quality and reliability. This precise level of labeling, surpasses the scope of publicly available datasets, bolstering our model more robust against common issues like overfitting and underfitting. Consequently, the system demonstrates greater efficacy and adaptability for real-world disease detection in diverse agricultural settings.

### Comprehensive evaluation of YOLO models for common bean disease detection

This study represents a trailblazing effort in evaluating one-stage YOLO object detectors, including YOLOv7, YOLOv8, and YOLO-NAS, specifically for detecting CB diseases. The YOLO series is known for its single-stage detection capability and real-time processing efficiency^68,69^. Notably, YOLO-NAS stands out within the YOLO family for its advanced performance in detection metrics and its rapid inference speed. We comprehensively assess the performance of our advanced YOLO-based detectors using a range of detailed metrics. The metrics encompass various annotation resolutions (whole and micro) for both leaf and pod datasets. This multifaceted evaluation approach allows us compare the detector’s performance across different plant parts, to providing a comprehensive analysis.

#### Training loss function

**Figure 5:**
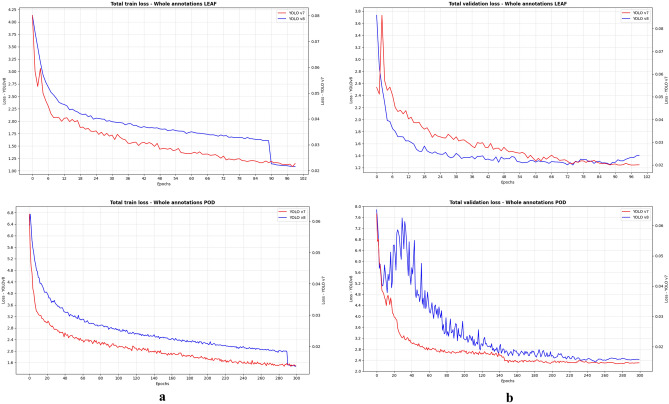
Total loss for different models, at whole annotations level; (**a**) train loss for leaves and pods, (**b**) validation loss for leaves and pods.

Training loss analysis plays a crucial role in emphasizing the efficiency, adaptability, and stability of the YOLOv7 and YOLOv8 models during the learning process. Both models exhibited a rapid initial decline in loss for both the leaf and pod datasets (see Fig. 5). This rapid decrease signifies the overall effectiveness of the learning and adaptation to the training data. This observation is consistent with prior findings on training duration and loss convergence^70^, affirming the diverse convergence rates observed during training. The consistent decline in training loss further validates the effectiveness of the model.

However, YOLOv8 model displayed an anomaly in the annotated pod dataset, where the loss starts to increase around epoch 16 before continuing on a downward trend. This could be attributed to the increased complexity of the annotated pod dataset compared to its previous training datasets. Once it overcome the initial hurdle, the model begins to effectively learn from the data, signifying a positive stability in the learning process.

Despite this anomaly, both models exhibited a consistent and steady decline in loss over time, indicating a positive stability in their learning process. Lower loss values on the training set compared to the validation set align with expectations. The relative stability in the difference between training and validation losses across epochs indicates the absence of significant overfitting in both the models, highlighting effective generalization, a common challenge in model training. This means that the models appear to learn underlying patterns in the data rather than memorizing specific training examples.

**Figure 6:**
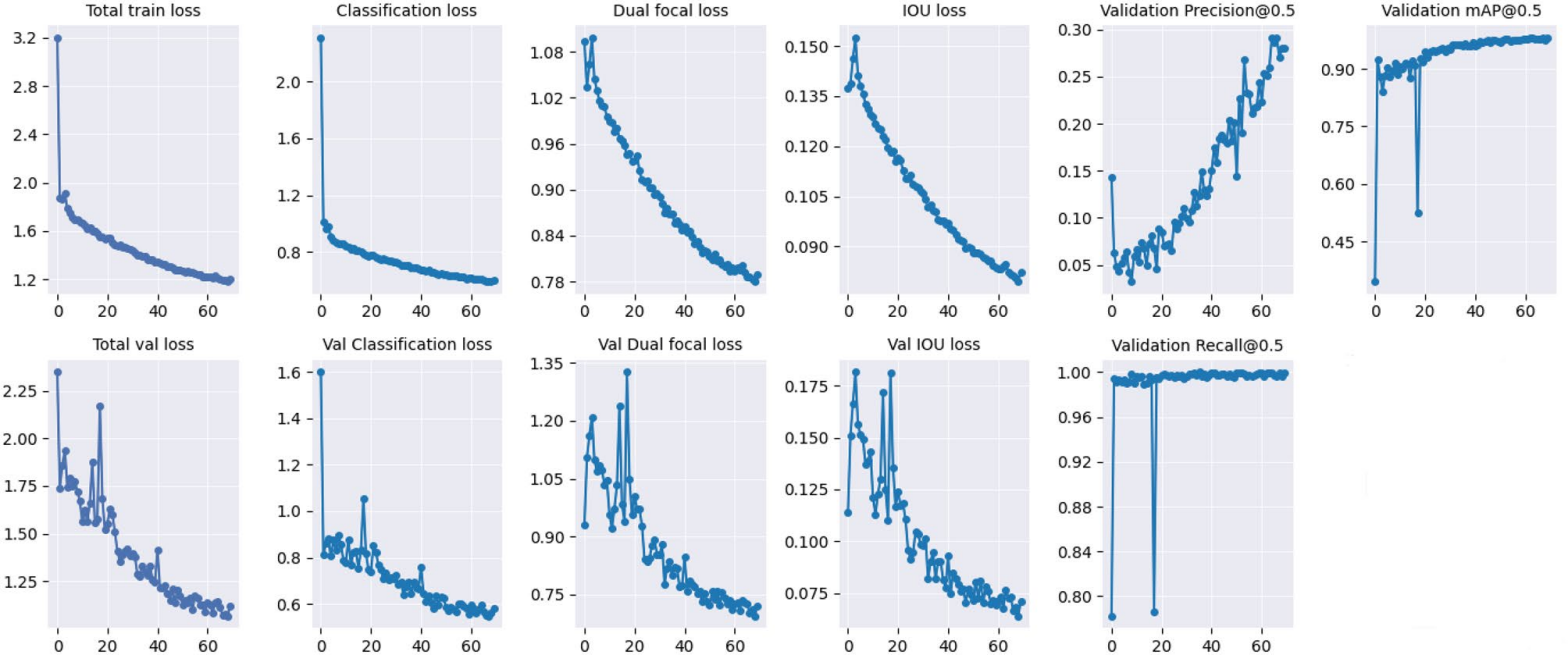
YOLO-NAS model evaluation indicators (loss and mAP@0.5) during training, for whole leaf annotations.

**Figure 7:**
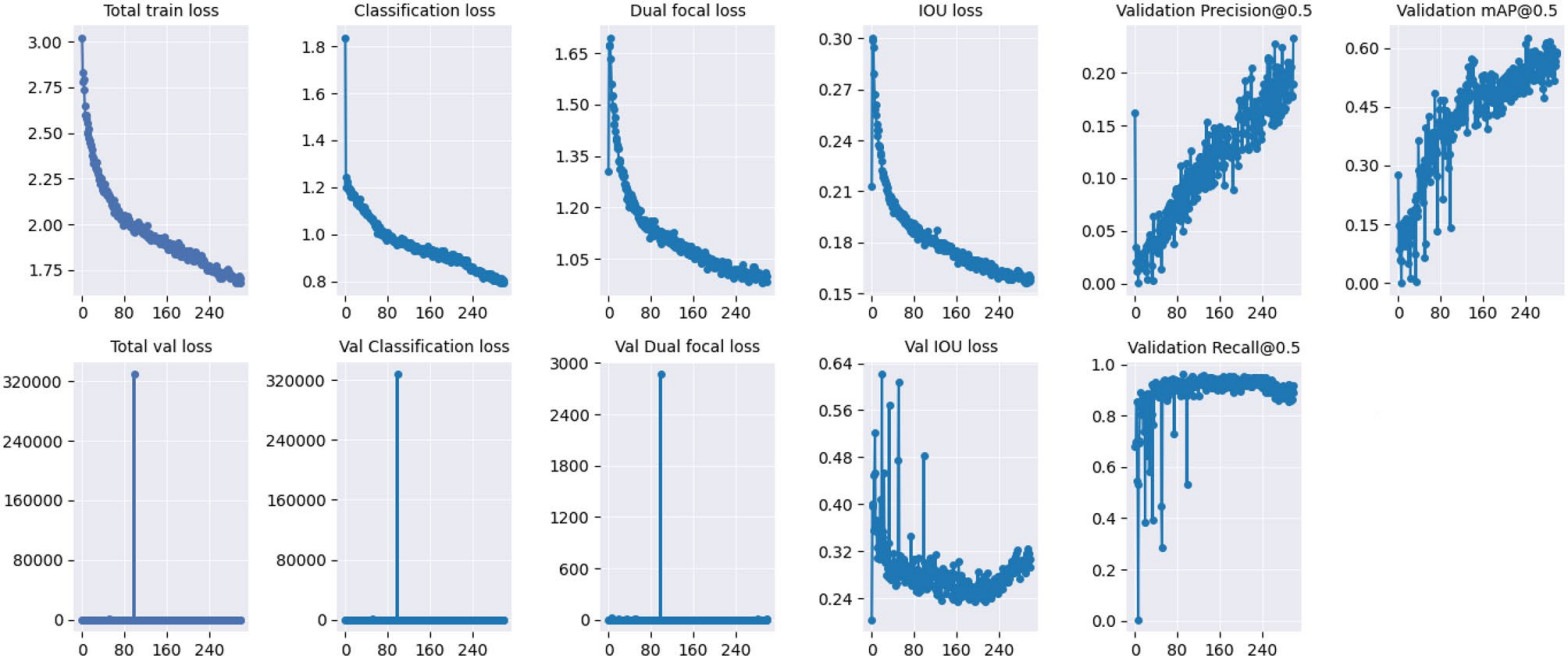
YOLO-NAS model evaluation indicators (loss and mAP@0.5) during training, for whole pod annotations.

On the contrary, while the YOLO-NAS model exhibited similar trends for the leaf dataset (Fig. 6), in both full annotation and micro-annotation levels (Supplementary Fig. 1), its validation losses for the pod dataset displayed significant fluctuations (Fig. 7, Supplementary Fig. 2). These fluctuations suggest potential overfitting, likely stemming from non-representative validation data or inadequate regularization techniques. This behavior could elucidate the lower mAP scores observed for YOLO-NAS in the pod dataset. It underscores the critical importance of careful dataset curation and the potential need for adjusting regularization while training such models.

Notably, at the micro annotation level, the YOLOv8 training on the pod dataset stopped at epoch 217 (Supplementary Fig. 3) due to the absence of improvement in the validation loss for 50 consecutive epochs. Additionally, YOLOv7 training on the leaf dataset showed a slight overfitting tendency as the validation loss followed an increasing trend around epoch 40 instead of decreasing, signifying an augmented difference between validation and training loss. The individual contributions of each loss to the total loss of the YOLOv7 model can be seen in Supplementary Figs. 4, 5, 6 and 7, and those of the YOLOv8 model in Supplementary Figs. 8, 9, 10 and 11.

#### mAP score

To assess the performance of object detection models, particularly focusing on the widely recognized mean Average Precision (mAP) metric^35^, we evaluated different YOLO models for leaf and pod detection. This metric has been the benchmark in competitions like PASCAL VOC, ImageNet, and COCO datasets. We complemented this analysis with confusion matrix to gain deeper insights into model performance, with a particular emphasis on unseen data at both whole and micro annotation levels.

The mAP scores of different YOLO models for leaf and pod detection were detailed in Table 3. YOLO-NAS stands out with a remarkable performance for whole leaf disease annotation, achieving an impressive mAP score of 97%. Nevertheless, for micro annotation on leaves, YOLOv8 excelled with a notable mAP score of 67%. In pod detection tasks, YOLOv8 continued to perform superior, achieving mAP scores of 96% and 87% for whole and micro annotations, respectively (Table 4). It is worth noting that YOLOv7 closely mirrored the performance of YOLOv8, achieving high mAP scores for both leaf and pod datasets.

**Table 3:** mAP metric score per leaf class for different models (IOU = 0.5, Conf = 0.35).

Classes	Whole annotations LEAF				Micro annotations LEAF			
	Images	mAP@0.5			Images	mAP@0.5		
		Yolo-NAS	YOLOv7	YOLOv8		Yolo-NAS	YOLOv7	YOLOv8
Healthy	986	0.958	0.968	0.85	986	0.958	0.963	0.98
ALS	1302	0.969	0.944	0.977	700	0.541	0.534	0.552
CBB	1430	0.969	0.955	0.943	700	0.668	0.548	0.697
CBA	1287	0.999	0.972	0.983	636	0.388	0.292	0.402
CBMV	1300	0.983	0.965	0.979	1300	0.975	0.948	0.971
BR	1088	0.997	0.997	0.995	654	0.453	0.358	0.437
Total	7393	0.979	0.967	0.955	4976	0.664	0.607	0.673

**Table 4:** mAP metric score per pod class for different models (IOU = 0.5, Conf = 0.35).

Classes	Whole annotations POD				Micro annotations POD			
	Images	mAP@0.5			Images	mAP@0.5		
		YOLO-NAS	YOLOv7	YOLOv8		YOLO-NAS	YOLOv7	YOLOv8
Healthy	1150	0.741	0.967	0.98	1000	0.681	0.96	0.987
ALS	1021	0.831	0.945	0.955	998	0.619	0.601	0.753
Total	2171	0.786	0.956	0.9675	1998	0.65	0.78	0.87

**Figure 8:**
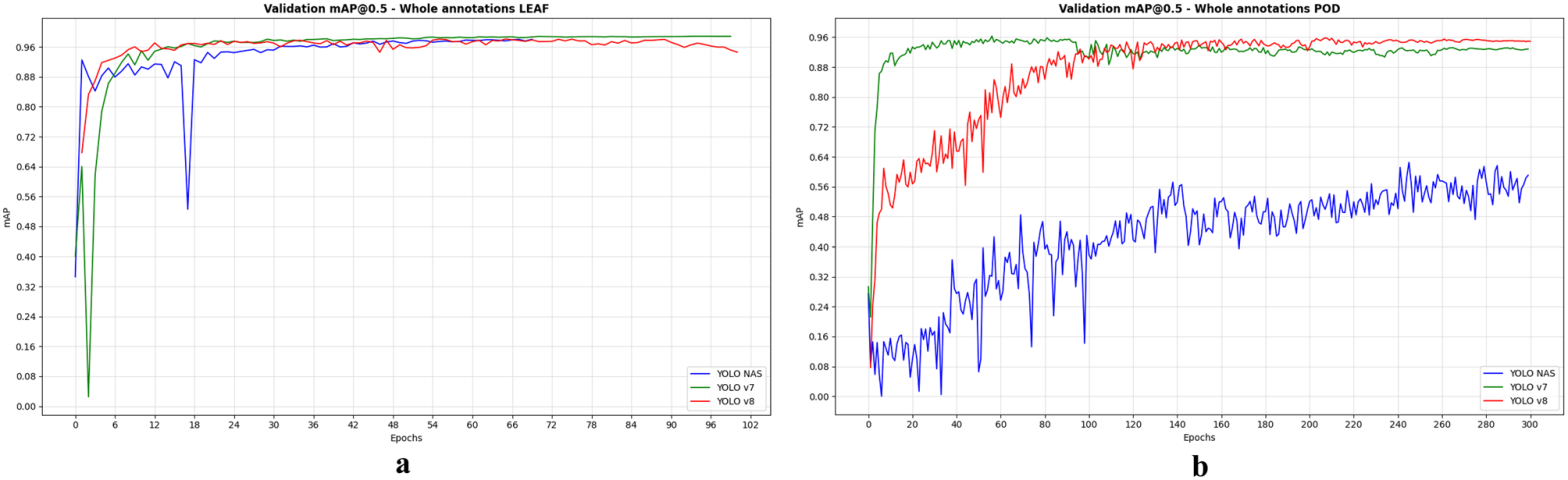
mAP@0.5 evaluation metric for different models; (**a**) whole leaf annotations, (**b**) whole pod annotations.

Across all classes and models, whole annotations generally yielded better results than micro annotations. Specifically, for the healthy class, YOLOv7 and YOLOv8 achieved high mAP accuracies of 96% and 98%, respectively, for pods across both annotation levels, and for leaves at the micro annotation level. In the case of whole leaf annotations, these models also performed well, with mAP scores of 96% and 85%, respectively. The evolution of the validation mAP during model training is illustrated in Fig. 8, demonstrating a continual increase of the mAP throughout the epochs until it becomes relatively uniform.

These findings provide further context to our study, emphasizing that although all three YOLO versions can achieve high accuracy, their performance nuances become apparent based on the complexity of annotation levels and the specific nature of the tasks. The noteworthy performance of YOLO-NAS under certain conditions and the close competition between YOLOv7 and YOLOv8 highlight the continuous advancements in object detection technologies, showcasing their potential applications in precision agriculture.

The differences in loss patterns and mAP scores among the models suggest that while YOLOv7 and YOLOv8 exhibit robustness in a various scenario, YOLO-NAS may require more specific tuning, especially when confronted with datasets of higher variability or complexity. This insight proves invaluable for future model development and application, particularly in precision agriculture, where precise and reliable disease detection is imperative. These findings underscore the necessity for continuous model evaluation and adjustment to cater to the specific characteristics of diverse datasets and detection tasks.

#### Performance of YOLO models using confusion matrix

**Figure 9:**
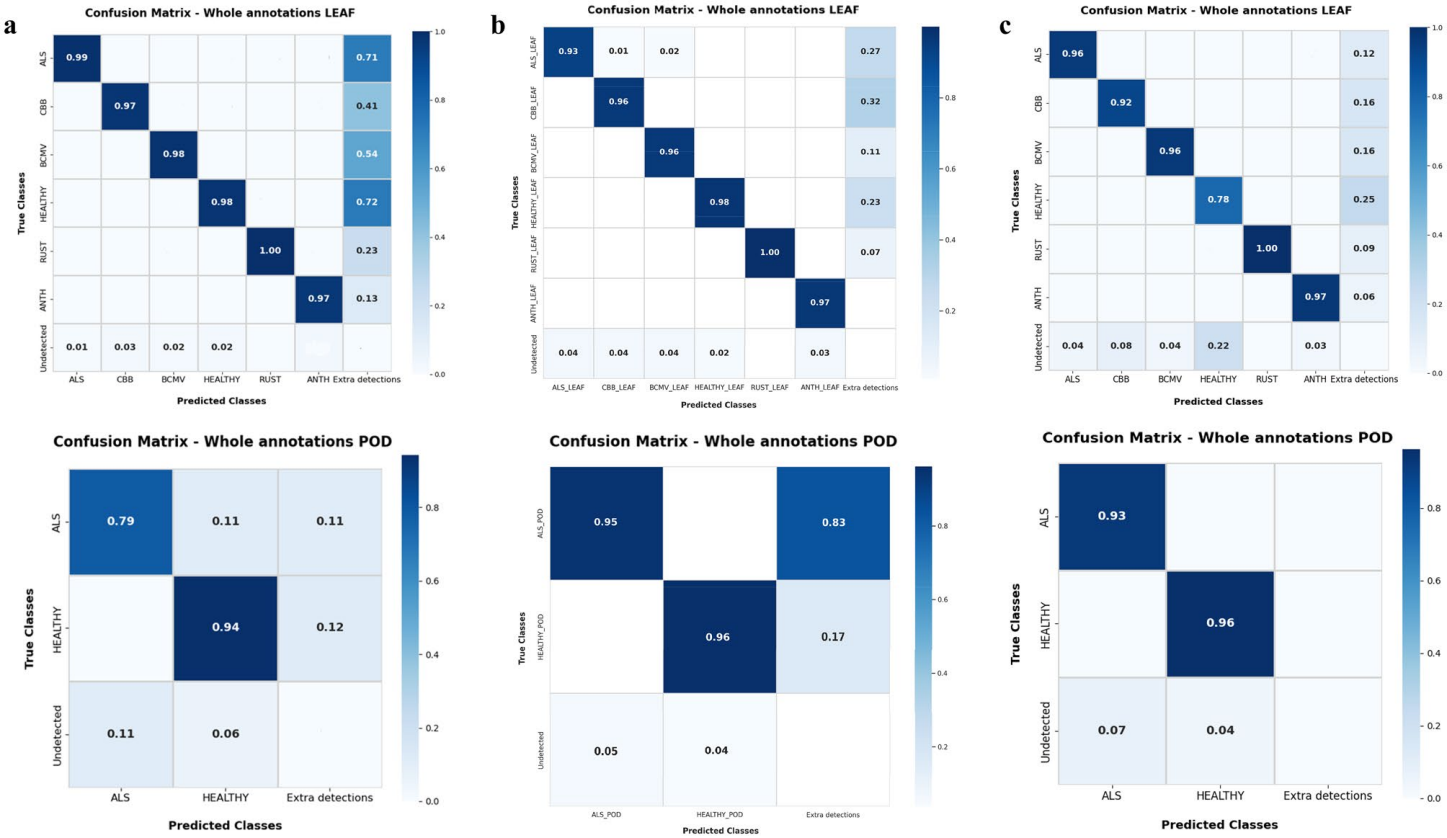
Confusion matrix using whole annotations for different models, (**a**) YOLO-NAS model, (**b**) YOLOv7 model, (**c**) YOLOv8 model.

In our study, confusion matrices served as a pivotal tool for assessing the performance of various YOLO model variants. These matrices, delineating true positives (TP), true negatives (TN), false positives (FP), and false negatives (FN), played a crucial role in evaluating disease-specific accuracy and identifying misclassifications (Fig. 9). The analysis revealed valuable instances, where class complexity resulted in reduced accuracy, providing insights into areas prone to errors for targeted improvements.

The YOLO-NAS model demonstrated robustness with over 97% accuracy in detecting symptoms of leaf diseases across all classes. However, for ALS pod symptoms, YOLOv7 and YOLOv8 outperformed YOLO-NAS with accuracies of 95% and 93%, respectively. YOLO-NAS’s detection rate for these symptoms dropped from 79% for whole annotations to 48% at the micro annotation level, compared to 66% each for YOLOv7 and YOLOv8. Diseases like rust and anthracnose, particularly challenging at micro levels, showed lower accuracies around 56%. No misclassifications occur at the micro annotation level (Supplementary Fig. 12), but the number of undetected objects increases considerably.

Interestingly, the confusion matrices highlighted additional detections made by the models. Despite these additional detections slightly affected precision, they were mostly correct and indicated the models’ effectiveness in identifying objects––a critical factor in complex agricultural scenarios where exhaustive annotation might be challenging. This aspect is significant as it showcases the models’ capability in comprehensive detection despite the inherent difficulties in annotating every detail in a small diseased image. Furthermore, recognizing the widespread occurrence of CB leaf spot disease across diverse regions such as Asia, Latin America, and Africa, we are actively compiling and annotating early-stage symptom images. This endeavor aims at enhancing models’ accuracy and adaptability for real agricultural settings.

#### Precision and recall

Our study also focused on evaluating the performance of various YOLO models in detecting CB diseases, utilizing precision and recall as key metrics. Precision, indicating the ratio of correctly identified positive cases to all predicted positives, and recall, measuring the ratio of correctly identified positives out of all actual positive cases, are essential metrics for assessing the diagnostic accuracy of the models.

The YOLOv7 and YOLOv8 models exhibited excellent performance, achieving precision and recall scores of 0.9 at the whole annotation level for both leaves and pods, as shown in Table 5. However, a decrease in these scores was observed for micro annotations, suggesting a variation in model performance based on annotation detail. YOLO-NAS demonstrated high effectiveness, particularly in whole-leaf annotations, with a precision of 0.7 and an impressive recall of 0.988. Despite its lower precision due to a higher number of extra detections, its high recall confirms its strong detection capability. For whole pod annotations, YOLO-NAS showed a precision of 0.6, primarily impacted by the model misclassifying some diseased pods as healthy (Fig. 9). The model maintained high recall levels even at the micro annotation level, though there was a slight drop compared to whole annotations.

**Table 5:** Precision and recall metrics score for different models (IOU = 0.5, Conf = 0.35).

Model	Whole annotations LEAF		Micro annotations LEAF		Whole annotations POD		Micro annotations POD	
	Precision	Recall	Precision	Recall	Precision	Recall	Precision	Recall
YOLO-NAS	0.706	0.988	0.508	0.713	0.625	0.918	0.567	0.637
YOLOv7	0.954	0.958	0.725	0.687	0.972	0.945	0.773	0.815
YOLOv8	0.962	0.925	0.774	0.566	0.99	0.936	0.848	0.818

**Figure 10:**
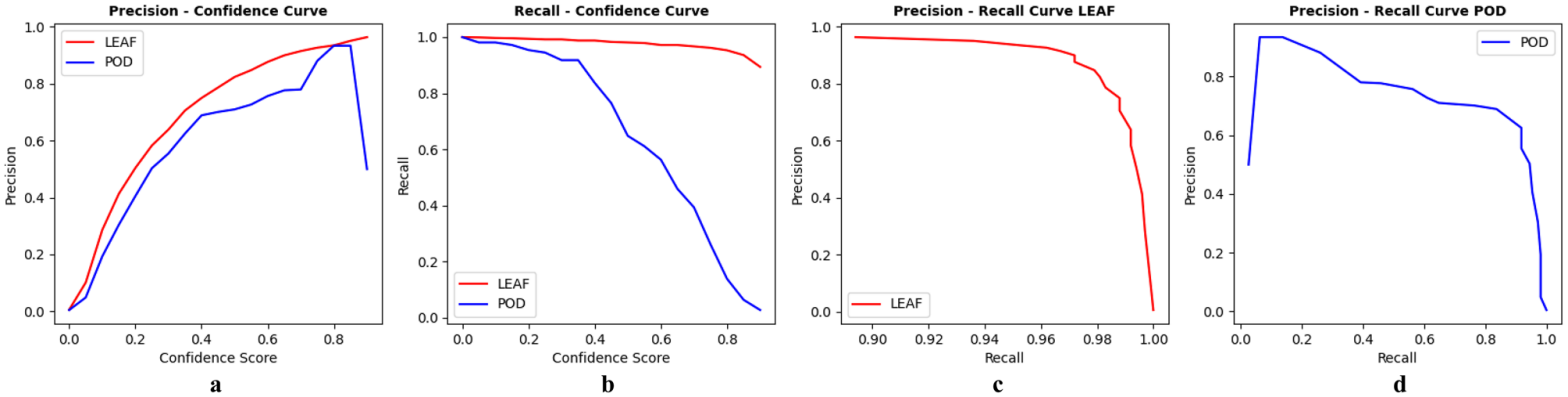
Operation results curve for YOLO-NAS model, using whole leaf and pod annotations; (**a**) precision-confidence curve, (**b**) recall-confidence curve, (**c**) precision-recall curve for leaf, and (**d**) precision-recall curve for pod.

In the evaluation of the performance of models, a confidence threshold (conf) of 0.35 was set uniformly across all three models. This threshold choice involves a trade-off between precision and recall, where a higher confidence threshold tends to increase precision by reducing false positives but may decrease recall as some true positives with lower confidence might be overlooked (Fig. 10a,b, Supplementary Figs. 13–20b,c). This delicate balance is visually represented in the Precision-Recall (PR) curve (Fig. 10c,d, Supplementary Figs. 13–20a), with the area under the PR curve (AUPRC) serving as a comprehensive metric summarizing of model performance across all thresholds. A similar behavior is observed at the micro annotation level (Supplementary Fig. 21).

The standardized dataset employed in our study ensures rigorous evaluation, enabling reliable comparisons of each model’s performance.

### Model predictions using unseen images

**Figure 11:**
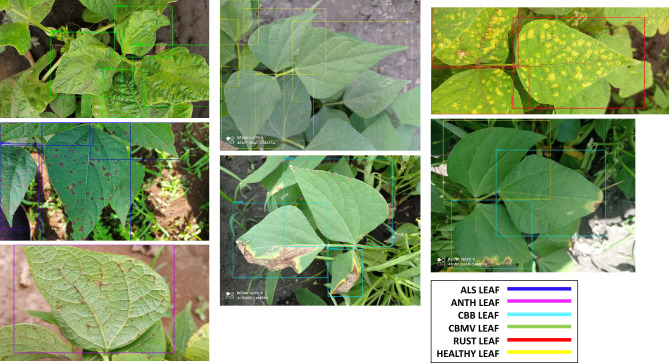
Some examples of common bean disease detection results using YOLO-NAS and whole annotations.

**Figure 12:**
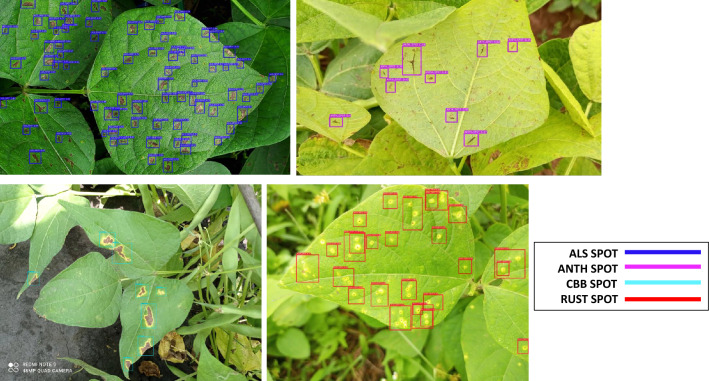
Some examples of common bean disease detection results using YOLO-NAS and micro annotations.

**Figure 13:**
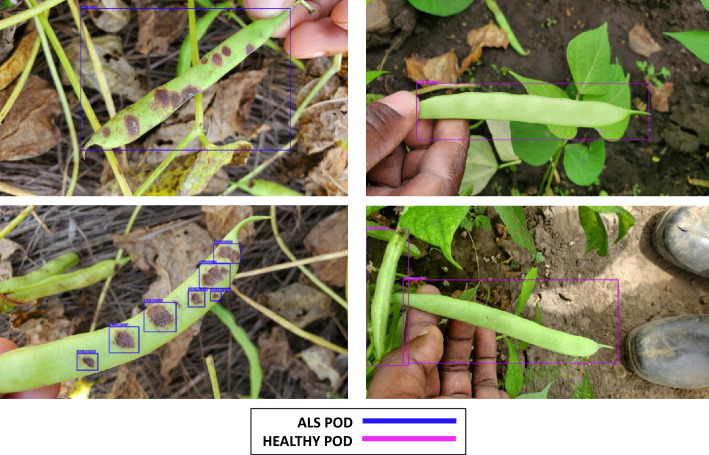
Some examples of common bean disease detection results using YOLO-NAS on the pod dataset.

After analyzing the results obtained from all the metrics mentioned above, Fig. 11 shows the predictions made by the YOLO-NAS model on the leaf dataset at the whole annotation level, Fig. 12 at the micro annotation level, and finally Fig. 13 shows the predictions on the pod dataset at both annotation levels.

Surprisingly, our analysis found that micro annotation yielded lower performance than whole annotation across all explored YOLO models, regardless of disease classes. This result contradicts the hypothesis that micro annotations will improve detection accuracy. The discrepancy suggests that the effectiveness of annotation methods may vary depending on factors such as dataset complexity, disease characteristics, and model requirements. However, the detection accuracy may be lower with micro annotation models because we did not fully annotate all lesions in each image, particularly due to the high number of lesions per image. Developing improved annotation techniques could significantly enhance the accuracy and efficiency of these annotations. Further investigation is warranted to understand this finding and optimize annotation strategies for future CB disease detection research.

**Figure 14:**
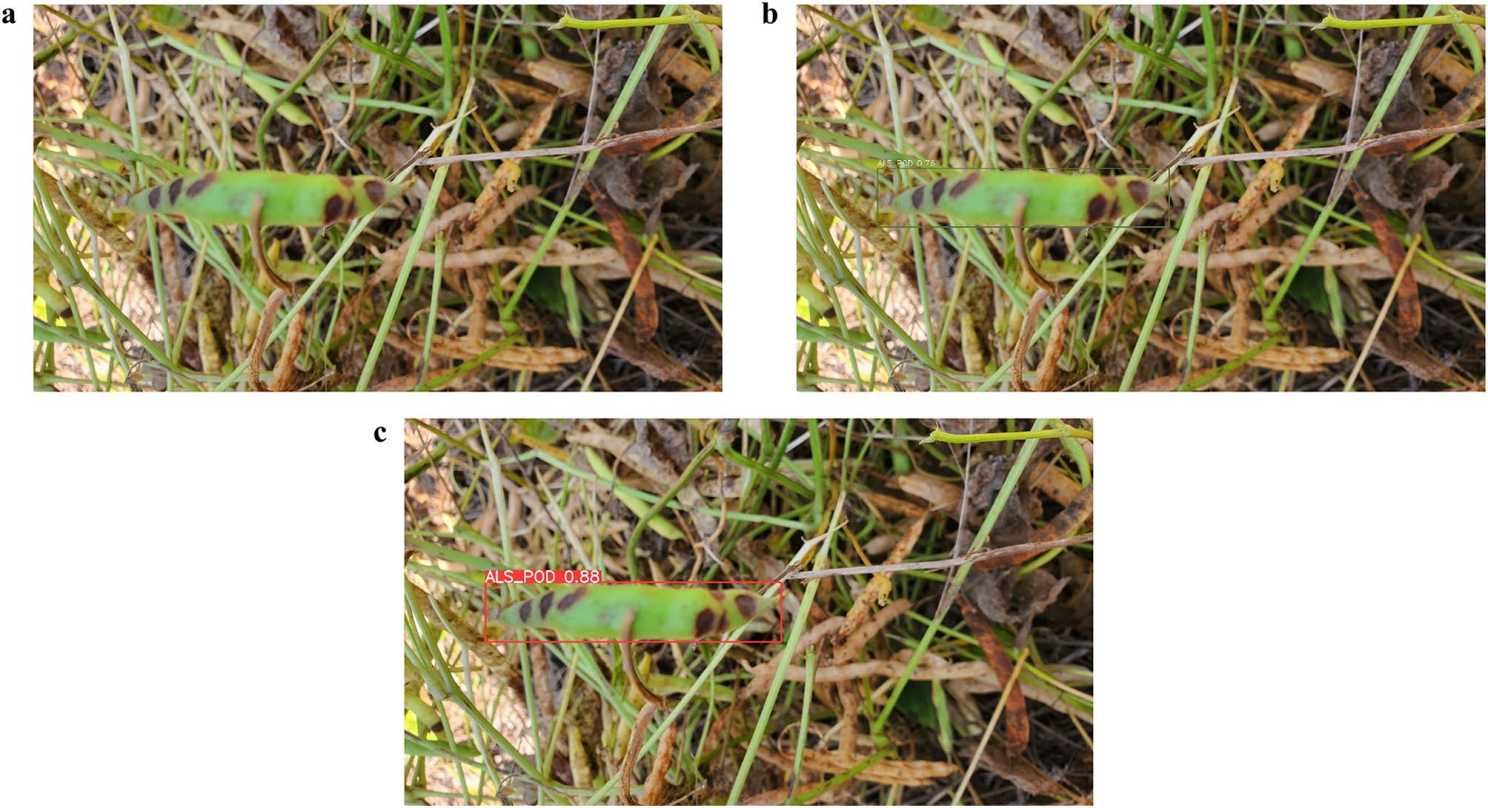
Example of prediction in the same image. (**a**) YOLO-NAS model, (**b**) YOLOv7 model, (**c**) YOLOv8 model.

**Figure 15:**
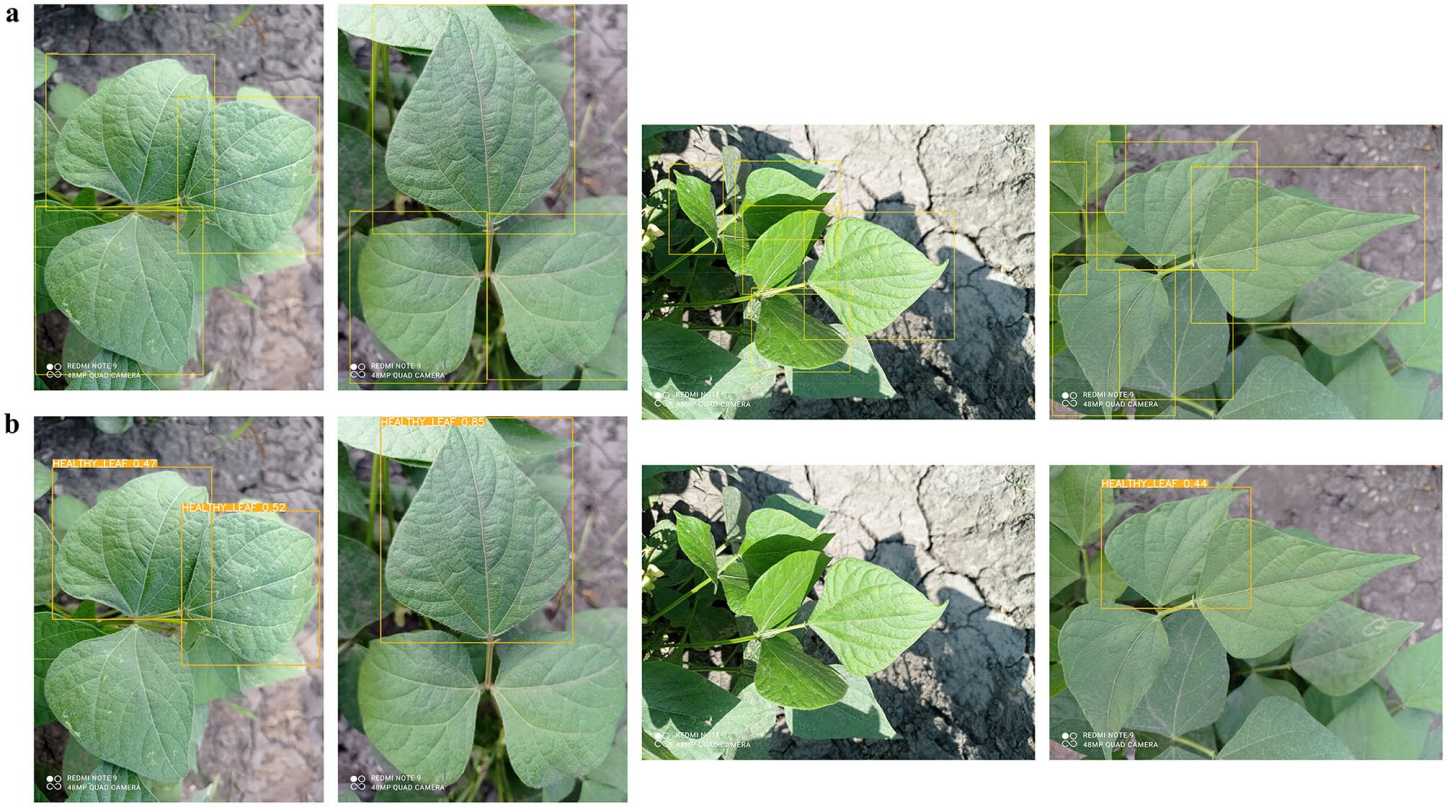
Example of prediction in the same image. (**a**) YOLO-NAS model, (**b**) YOLOv8 model.

Conversely, Figs. 14 and 15 show situations where specific model outperform others. For example, in Fig. 14, the YOLOv7 and YOLOv8 models successfully detect the POD within the image, whereas YOLO-NAS does not. This aligns with the mAP, precision, and recall results as shown above. However, Fig. 15, demonstrates YOLO-NAS ability to more accurately identify healthy leaves, while the YOLOv8 model fails to detect many of the healthy leaves. This aligns with the model’s respective mAP scores for the healthy class.

### Deployment and testing of the YOLO-NAS model in an AI powered app

**Figure 16:**
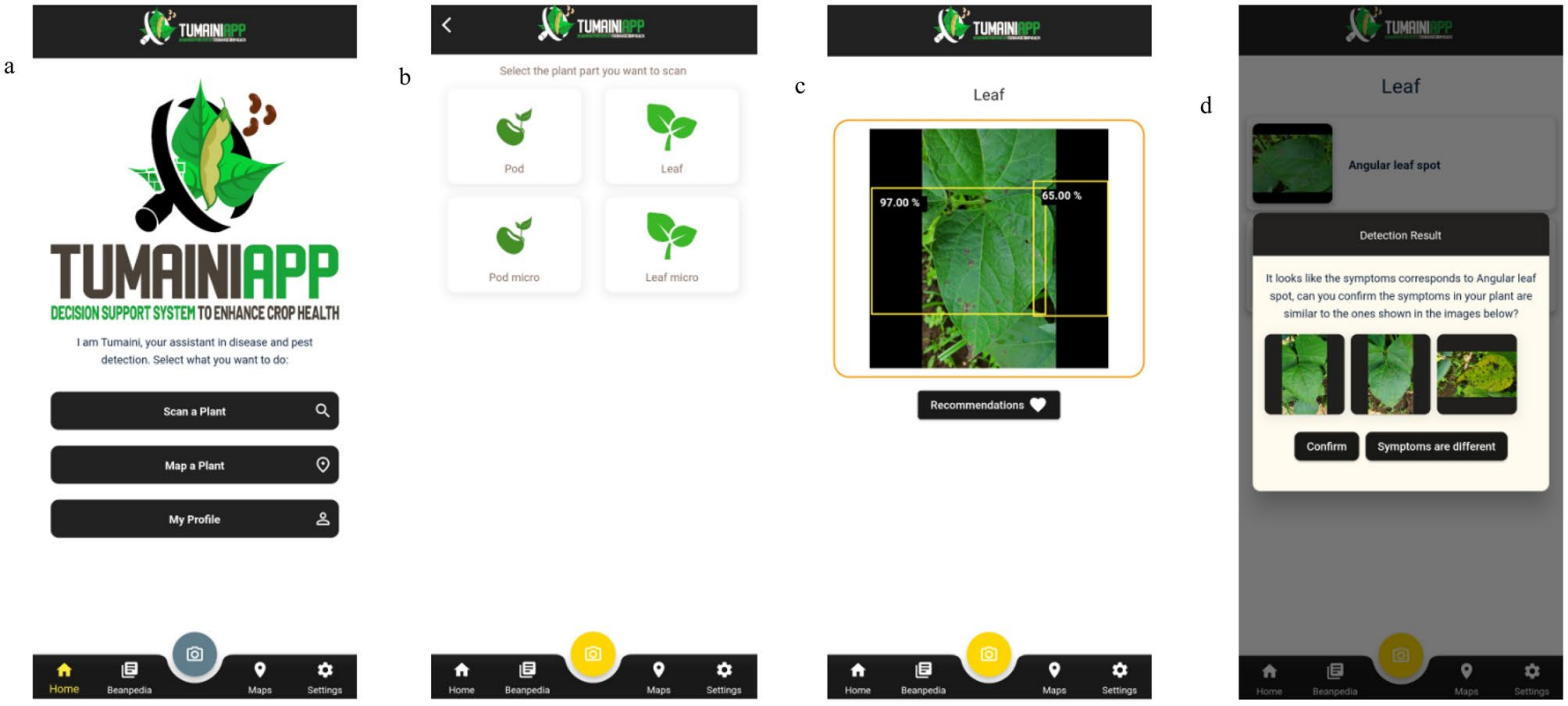
Developed mobile application for bean disease detection. (**a**) Initial screen, (**b**) image taking and scan, (**c**) diagnostic screen for leaf, (**d**) recommendations screen.

To bridge the gap between research and practical application, we seamlessly integrated our promising whole annotation YOLO-NAS models into a user-friendly Android app. The app boasts a straightforward design, allowing users to either upload existing photos from their storage or capture new ones in real time for immediate analysis (Fig. 16). This real-time capability played a pivotal role in evaluating the functional accuracy of the models within the practical context of a real-world app.

To evaluate the real-time performance of the app, we tested real-field images from disease hotspots in Latin America and Africa. Our results demonstrate that the YOLO-NAS model achieved outstanding accuracy in accurately detecting almost all classes (Table 6). Specifically, the model achieved close to 100% accuracy with high confidence scores across all disease classes, except for the pod classes (Table 6). The lower confidence in pod predictions can be attributed to the inherent complexity of the task. In real-field environments, bean pods are often surrounded by diverse background elements that can pose challenges to distinguish between bean pods and background elements, leading to lower confidence levels in its predictions. Furthermore, factors such as varying lighting conditions, shadows, and cluttered backgrounds can contribute to the difficulty of the task. Despite these limitations, the model successfully manages to detect almost all pods correctly.

**Table 6:** Real-time disease detection using the Tumaini AI app and validation.

Part	Class	# Images	Misdetections	Accuracy (%)	Average confidence (%)
Leaf	Healthy	50	0	100	95.88
	ALS	50	0	100	96.8
	CBB	50	0	100	93.88
	Anthracnose	50	0	100	96.38
	CBMV	50	0	100	97.76
	Rust	50	0	100	97.4
Pod	Healthy	50	3	94	69.8
	ALS	50	2	96	60.21

This successful field deployment not only validates the reliability of the app but also underscores its potential as a robust agricultural tool for enabling timely interventions in CB disease management practices that can significantly enhance crop yields and reduce reliance of pesticides.

### Advantages of YOLO-NAS models in precision agriculture

Our extensive analysis revealed the prowess of YOLO-NAS model in real-time CB disease detection within agricultural settings (Supplementary Fig. 22, Table 6). Engineered through the cutting-edge technique of Neural Architecture Search (NAS), YOLO-NAS adeptly strikes a balance between speed and accuracy amidst the challenges in field conditions, which is a pivotal attribute for prompt and precise disease diagnosis^71^. Notably, it effectively combines the quick detection characteristic of one-stage detectors and the precision akin to two-stage detectors, achieving high efficiency and precision, with reduced risk of overfitting^72^. The performance metrics of YOLO-NAS stands out for its exceptional performance, particularly for its high mAP scores on benchmark datasets like COCO and its lower latency compared to counterparts such as YOLOv7 and YOLOv8.

In a continuation of exploring the model performance, YOLOv7 and YOLOv8 models have demonstrated robustness for edge computing applications in agricultural contexts, especially in remote areas with limited internet connectivity. Our findings demonstrated high accuracy, precision, and recall achieved by these models, proving them reliable tools for rapid and effective CB disease management. Their ability to function independently on local devices empowers farmers to conduct immediate on-site diagnostics. This aligns with the findings^44^ which emphasized the proficiency of the YOLOv7 model in tea leaf disease detection. Their research, utilizing key metrics such as precision and recall, recorded values exceeding 90%, reinforces the effectiveness of YOLOv7 in accurately identifying plant diseases, mirroring our own observations with both YOLOv7 and YOLOv8 models.

In contrast, while YOLO-NAS exhibits good precision and generalization, its current complexity introduces challenges for offline use in the field conditions. This primarily stems from the lack of readily available tools to efficiently convert the model into a lightweight format suitable for mobile devices. This limitation renders YOLO-NAS as less viable for immediate use in the field without cloud support. Nevertheless, the ongoing advancements in YOLO models, particularly the online proficiency of YOLO-NAS, paint a promising future. Researchers aim to amalgamate the accuracy of YOLO-NAS with the offline capabilities of YOLOv7 and YOLOv8, expanding the accessibility of AI-powered disease detection tools to agricultural professionals worldwide, irrespective of their internet connectivity. This transformative interaction holds the potential to revolutionize disease management strategies in agriculture, offering a seamless blend of precision and accessibility.

## Conclusions and future directions

In conclusion, our research represents a significant stride in applying Deep Learning (DL) models for real-time crop disease detection under diverse field conditions. We achieved this by leveraging advanced deep transfer learning techniques, to empower real-time disease classification in CB. At the core of this innovative approach lie the YOLO architectures, notably YOLO-NAS, YOLOv7, and YOLOv8. These models serve a dual purpose: functioning as a diagnostic tool and serving as a comprehensive repository for CB data. These models successfully automated the identification of five major CB diseases, successfully distinguishing between healthy and diseased pods and leaves.

The outcomes are promising, with classification accuracy exceeding 95% for leaves and 78% for pods, accompanied by high precision and recall rates. Our extensive database, containing over 9500 images and 44,000 annotations, fuels the continuous refinement of these models and reflects our dedication to the ongoing development of TUMAINI AI Network (https://www.tumainiaiapp.org/). A significant outcome is the practical application of these models for early disease detection and management in CB. The validated AI app developed as part of this research empowers farmers, bean breeders, and agronomists with a robust decision support system, ultimately enhancing disease management strategies.

This research establishes a strategic foundation for integrating AI into CB disease prevention and management. By deploying validated YOLO-NAS models with an Android-based mobile AI application, tested with images from disease hotspots, we aim to make disease management intuitive and accessible for farmers. This tool targets improvements in agricultural practices, leading to higher productivity and sustainability in key regions like Latin America and Africa. Looking ahead, we envision AI not only forecasting but also actively participating in managing crop diseases, ultimately transforming agricultural practices, and contributing to crop production sustainability, worldwide. This work lays the groundwork for future innovations, solidifying AI’s role as a crucial tool in advancing agricultural resilience and early warning systems.

### Supplementary Information

Supplementary Figures.

Supplementary Tables.

## Data Availability

The datasets used and analyzed during the current study are available from the corresponding author upon request.

## References

[1] Nadeem MA (2021). Common bean as a potential crop for future food security: An overview of past, current and future contributions in genomics, transcriptomics, transgenics and proteomics. Biotechnol. Biotechnol. Equip..

[2] Huertas R, William Allwood J, Hancock RD, Stewart D (2022). Iron and zinc bioavailability in common bean (*Phaseolus vulgaris*) is dependent on chemical composition and cooking method. Food Chem..

[3] Petry N, Boy E, Wirth JP, Hurrell RF (2015). Review: The potential of the common bean (*Phaseolus vulgaris*) as a vehicle for iron biofortification. Nutrients.

[4] Nchanji EB, Lutomia CK, Chirwa R, Templer N, Rubyogo JC, Onyango P (2021). Immediate impacts of COVID-19 pandemic on bean value chain in selected countries in sub-Saharan Africa. Agric. Syst..

[5] Fininsa C, Yuen J (2001). Association of bean rust and common bacterial blight epidemics with cropping systems in Hararghe highlands, eastern Ethiopia. Int. J. Pest Manag..

[6] Aytenfsu M, Terefe H, Ayana G (2019). Distribution and association of common bean angular leaf spot (*Phaeoisariopsis griseola*) with biophysical factors in Southern and Southwestern Ethiopia. East Afr. J. Sci..

[7] Karavina, C., Mandumbu, R., Parwada, C. & Zivenge, E. *Epiphytic Survival of Xanthomonas axonopodis pv. phaseoli (E. F. SM)*. http://www.biosciences.elewa.org/JAPS;ISSN2071-7024 (2011).

[8] Landeras E, Trapiello E, Braña M, González AJ (2017). Occurrence of angular leaf spot caused by *Pseudocercospora griseola* in *Phaseolus vulgaris* in Asturias, Spain. Span. J. Agric. Res..

[9] Kennelly M, O’Mara J, Rivard C, Miller GL, Smith D (2012). Introduction to abiotic disorders in plants. Plant Health Instruct..

[10] Talaviya T, Shah D, Patel N, Yagnik H, Shah M (2020). Implementation of artificial intelligence in agriculture for optimisation of irrigation and application of pesticides and herbicides. Artif. Intell. Agric..

[11] Javaid M, Haleem A, Singh RP, Suman R (2022). Enhancing smart farming through the applications of Agriculture 4.0 technologies. Int. J. Intell. Netw..

[12] Javaid M, Haleem A, Khan IH, Suman R (2023). Understanding the potential applications of artificial intelligence in agriculture sector. Adv. Agrochem..

[13] Adli HK (2023). Recent advancements and challenges of AIoT application in smart agriculture: A review. Sensors.

[14] Ramcharan A (2019). A mobile-based deep learning model for cassava disease diagnosis. Front. Plant Sci..

[15] Tang Z, Yang J, Li Z, Qi F (2020). Grape disease image classification based on lightweight convolution neural networks and channelwise attention. Comput. Electron. Agric..

[16] Gomez Selvaraj M (2020). Detection of banana plants and their major diseases through aerial images and machine learning methods: A case study in DR Congo and Republic of Benin. ISPRS J. Photogramm. Remote Sens..

[17] Siddiqua A, Kabir MA, Ferdous T, Ali IB, Weston LA (2022). Evaluating plant disease detection mobile applications: Quality and limitations. Agronomy.

[18] Waheed H, Akram W, Ul Islam S, Hadi A, Boudjadar J, Zafar N (2023). A mobile-based system for detecting ginger leaf disorders using deep learning. Future Internet.

[19] Khan AT, Jensen SM, Khan AR, Li S (2023). Plant disease detection model for edge computing devices. Front. Plant Sci..

[20] Mendes J (2020). Smartphone applications targeting precision agriculture practices—A systematic review. Agronomy.

[21] Al-Adhaileh MH, Aldhyani THH (2022). Artificial intelligence framework for modeling and predicting crop yield to enhance food security in Saudi Arabia. PeerJ Comput. Sci..

[22] Altalak M, Uddin MA, Alajmi A, Rizg A (2022). Smart agriculture applications using deep learning technologies: A survey. Appl. Sci..

[23] Dhanya VG (2022). Deep learning based computer vision approaches for smart agricultural applications. Artif. Intell. Agric..

[24] Khalid, A., Akbar, S., Hassan, S. A., Firdous, S. & Gull, S. Detection of tomato leaf disease using deep convolutional neural networks. In *2023 4th International Conference on Advancements in Computational Sciences, ICACS 2023—Proceedings*. 10.1109/ICACS55311.2023.10089689 (2023).

[25] Jiang F, Lu Y, Chen Y, Cai D, Li G (2020). Image recognition of four rice leaf diseases based on deep learning and support vector machine. Comput. Electron. Agric..

[26] Lu Y, Yi S, Zeng N, Liu Y, Zhang Y (2017). Identification of rice diseases using deep convolutional neural networks. Neurocomputing.

[27] Sudhesh KM, Sowmya V, SainamoleKurian P, Sikha OK (2023). AI based rice leaf disease identification enhanced by dynamic mode decomposition. Eng. Appl. Artif. Intell..

[28] Johannes A (2017). Automatic plant disease diagnosis using mobile capture devices, applied on a wheat use case. Comput. Electron. Agric..

[29] Nigam S (2023). Deep transfer learning model for disease identification in wheat crop. Ecol. Inform..

[30] Haque MA (2022). Deep learning-based approach for identification of diseases of maize crop. Sci. Rep..

[31] Durmus, H., Gunes, E. O. & Kirci, M., Disease detection on the leaves of the tomato plants by using deep learning. In *2017 6th International Conference on Agro-Geoinformatics, Agro-Geoinformatics 2017*. 10.1109/AGRO-GEOINFORMATICS.2017.8047016 (2017).

[32] Brahimi M, Boukhalfa K, Moussaoui A (2017). Deep learning for tomato diseases: Classification and symptoms visualization. Appl. Artif. Intell..

[33] Fuentes A, Yoon S, Kim SC, Park DS (2017). A robust deep-learning-based detector for real-time tomato plant diseases and pests recognition. Sensors.

[34] Shoaib M (2022). Deep learning-based segmentation and classification of leaf images for detection of tomato plant disease. Front. Plant Sci..

[35] Selvaraj MG (2019). AI-powered banana diseases and pest detection. Plant Methods.

[36] Liu B, Ding Z, Tian L, He D, Li S, Wang H (2020). Grape leaf disease identification using improved deep convolutional neural networks. Front. Plant Sci..

[37] Çetiner H (2022). Citrus disease detection and classification using based on convolution deep neural network. Microprocess. Microsyst..

[38] Dhiman P (2022). A novel deep learning model for detection of severity level of the disease in citrus fruits. Electronics.

[39] da Silva JCF, Silva MC, Luz EJS, Delabrida S, Oliveira RAR (2023). Using mobile edge AI to detect and map diseases in citrus orchards. Sensors.

[40] Mia MdR, Roy S, Das SK, Rahman MdA (2020). Mango leaf disease recognition using neural network and support vector machine. Iran J. Comput. Sci..

[41] Rahaman N (2023). A deep learning based smartphone application for detecting mango diseases and pesticide suggestions. Int. J. Comput. Dig. Syst..

[42] Hu G, Wang H, Zhang Y, Wan M (2021). Detection and severity analysis of tea leaf blight based on deep learning. Comput. Electr. Eng..

[43] Bao W, Fan T, Hu G, Liang D, Li H (2022). Detection and identification of tea leaf diseases based on AX-RetinaNet. Sci. Rep..

[44] Soeb MJA (2023). Tea leaf disease detection and identification based on YOLOv7 (YOLO-T). Sci. Rep..

[45] Zhang J, Rao Y, Man C, Jiang Z, Li S (2021). Identification of cucumber leaf diseases using deep learning and small sample size for agricultural Internet of Things. Int. J. Distrib. Sens. Netw..

[46] Khan MA (2022). Cucumber leaf diseases recognition using multi level deep entropy-ELM feature selection. Appl. Sci..

[47] Yigezu, M. G., Woldeyohannis, M. M. & Tonja, A. L. Early ginger disease detection using deep learning approach. In *Lecture Notes of the Institute for Computer Sciences, Social-Informatics and Telecommunications Engineering, LNICST* 411 480–488. 10.1007/978-3-030-93709-6_32/COVER (2022).

[48] Narmilan A, Gonzalez F, Surantha A, Salgadoe A, Powell K (2022). Detection of white leaf disease in sugarcane using machine learning techniques over UAV multispectral images. Drones.

[49] Kumpala I, Wichapha N, Prasomsab P (2022). Sugar cane red stripe disease detection using YOLO CNN of deep learning technique. Eng. Access.

[50] de Moraes JL, de Oliveira Neto J, Badue C, Oliveira-Santos T, de Souza AF (2023). Yolo-Papaya: A papaya fruit disease detector and classifier using CNNs and convolutional block attention modules. Electronics.

[51] Coulibaly S, Kamsu-Foguem B, Kamissoko D, Traore D (2019). Deep neural networks with transfer learning in millet crop images. Comput. Ind..

[52] Bari BS (2021). A real-time approach of diagnosing rice leaf disease using deep learning-based faster R-CNN framework. PeerJ Comput. Sci..

[53] Kalidindi, L. D. & Vijayabaskar, V. Plant disease detection using faster RCNN networks. In *Proc.—2022 International Conference on Computing, Communication and Power Technology, IC3P 2022* 260–263. 10.1109/IC3P52835.2022.00062 (2022).

[54] Nawaz M, Nazir T, Javed A, Tawfik Amin S, Jeribi F, Tahir A (2024). CoffeeNet: A deep learning approach for coffee plant leaves diseases recognition. Expert Syst. Appl..

[55] Liu, N. & Han, J. *DHSNet: Deep Hierarchical Saliency Network for Salient Object Detection* 678–686 (2016).

[56] Nasution SW, Kartika K (2022). Eggplant disease detection using yolo algorithm telegram notified. Int. J. Eng. Sci. Inf. Technol..

[57] Wang Z, Xie Q, Wei M, Long K, Wang J (2022). Multi-feature fusion VoteNet for 3D object detection. ACM Trans. Multimedia Comput. Commun. Appl..

[58] Zeng S, Yang W, Jiao Y, Geng L, Chen X (2023). SCA-YOLO: A new small object detection model for UAV images. Vis. Comput..

[59] Ouf NS (2023). Leguminous seeds detection based on convolutional neural networks: Comparison of faster R-CNN and YOLOv4 on a small custom dataset. Artif. Intell. Agric..

[60] Slimani H, El Mhamdi J, Jilbab A (2024). Advancing disease identification in fava bean crops. J. Intell. Fuzzy Syst..

[61] Paszke, A. *et al.* PyTorch: An imperative style, high-performance deep learning library. *Adv. Neural. Inf. Process Syst*. **32** (2019).

[62] Terven J, Cordova-Esparza D (2023). A comprehensive review of YOLO architectures in computer vision: From YOLOv1 to YOLOv8 and YOLO-NAS. Mach. Learn. Knowl. Extr..

[63] *Deci-AI Official Repository—Super Gradients*. https://github.com/Deci-AI/super-gradients/blob/master/YOLONAS.md.

[64] Reis, D., Kupec, J., Hong, J. & Daoudi, A. *Real-Time Flying Object Detection with YOLOv8*. https://arxiv.org/abs/2305.09972v1 (2023).

[65] *Ultralytics Official Repository*. https://github.com/ultralytics/ultralytics.

[66] Wang, C.-Y., Bochkovskiy, A. & Liao, H.-Y. M. *YOLOv7: Trainable Bag-of-Freebies Sets New State-of-the-Art for Real-Time Object Detectors* 7464–7475. 10.1109/cvpr52729.2023.00721 (2022).

[67] Rodríguez De Luque JJ, Creamer B (2014). Principales restricciones y tendencias en la producción y comercialización de fríjol común; estableciendo prioridades de investigación. Agron. Colomb..

[68] Diwan T, Anirudh G, Tembhurne JV (2023). Object detection using YOLO: Challenges, architectural successors, datasets and applications. Multimed. Tools Appl..

[69] Qureshi, R. *et al. A Comprehensive Systematic Review of YOLO for Medical Object Detection (2018 to 2023)*. 10.36227/TECHRXIV.23681679.V1 (2023).

[70] Zhao, H. *et al.* Real-time object detection and robotic manipulation for agriculture using a YOLO-based learning approach. http://arxiv.org/abs/2401.15785 (2024).

[71] Casas E, Ramos L, Bendek E, Rivas-Echeverria F (2023). Assessing the effectiveness of YOLO architectures for smoke and wildfire detection. IEEE Access.

[72] Badgujar, C.M., Poulose, A. & Gan, H. *Agricultural Object Detection with You Look Only Once (YOLO) Algorithm: A Bibliometric and Systematic Literature Review*. https://arxiv.org/abs/2401.10379v1 (2024).

