# Supplementary material for: Advancing common bean (Phaseolus vulgaris L.) disease detection with YOLO driven deep learning to enhance agricultural AI
Source: Sci Rep. 2024 Jul 6;14:15596. doi: 10.1038/s41598-024-66281-w (PMC11227504; doi:10.1038/s41598-024-66281-w)
Supplement: Supplementary file 1 — Supplementary Figures. [file 41598_2024_66281_MOESM1_ESM.pptx]

## Slide 1
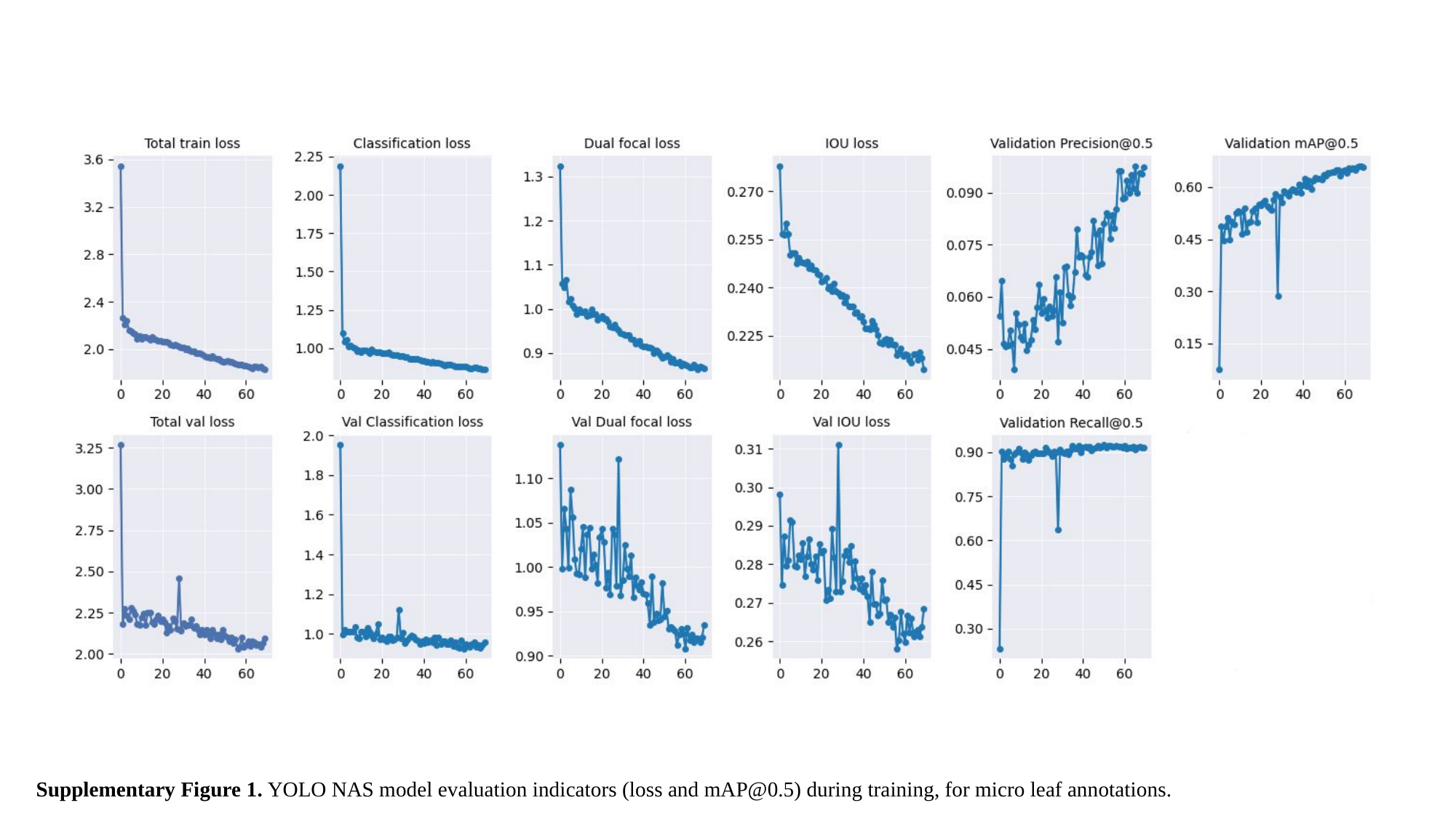

Supplementary Figure 1. YOLO NAS model evaluation indicators (loss and mAP@0.5) during training, for micro leaf annotations.

## Slide 2
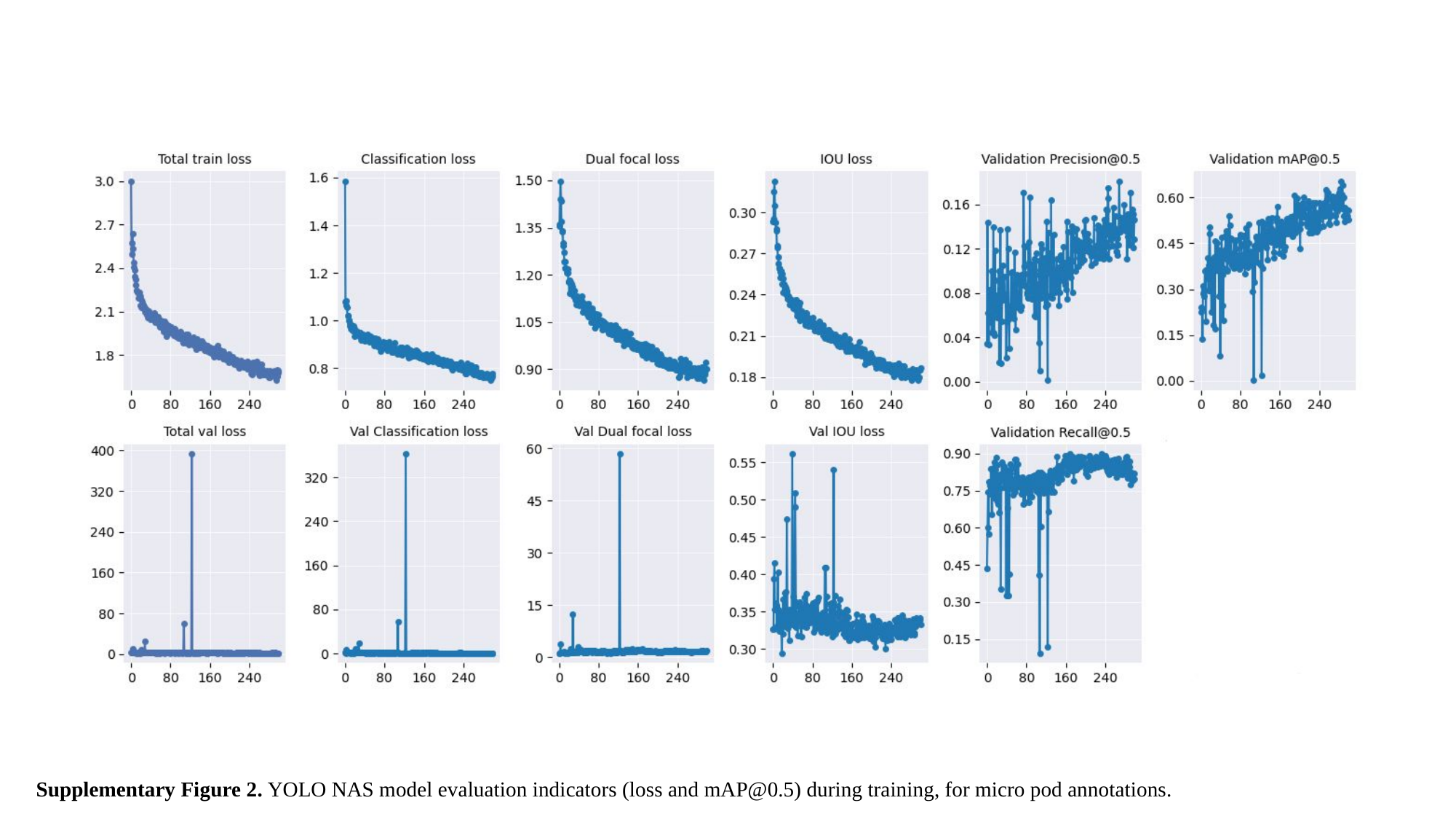

Supplementary Figure 2. YOLO NAS model evaluation indicators (loss and mAP@0.5) during training, for micro pod annotations.

## Slide 3
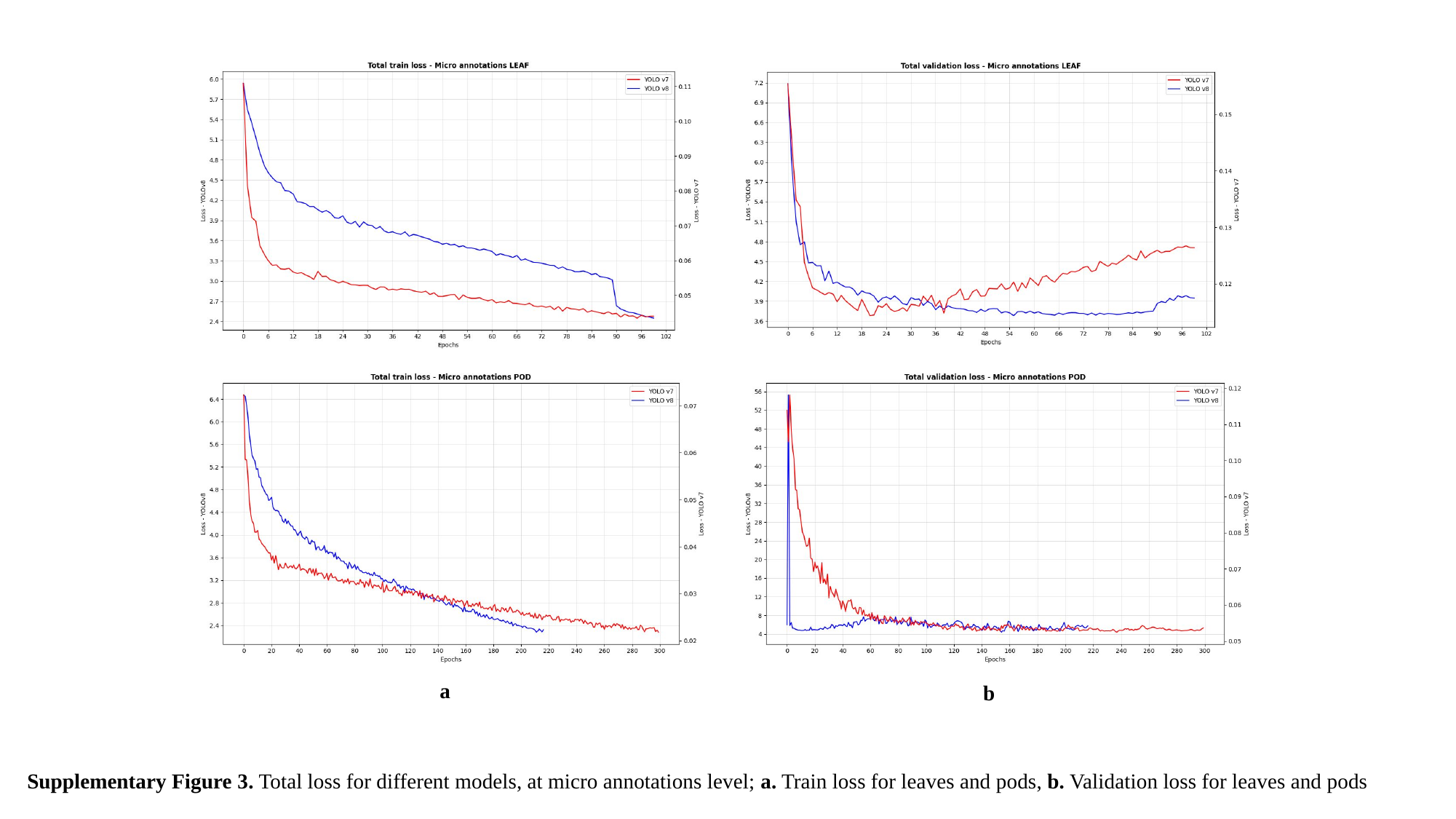

a
b
Supplementary Figure 3. Total loss for different models, at micro annotations level; a. Train loss for leaves and pods, b. Validation loss for leaves and pods

## Slide 4
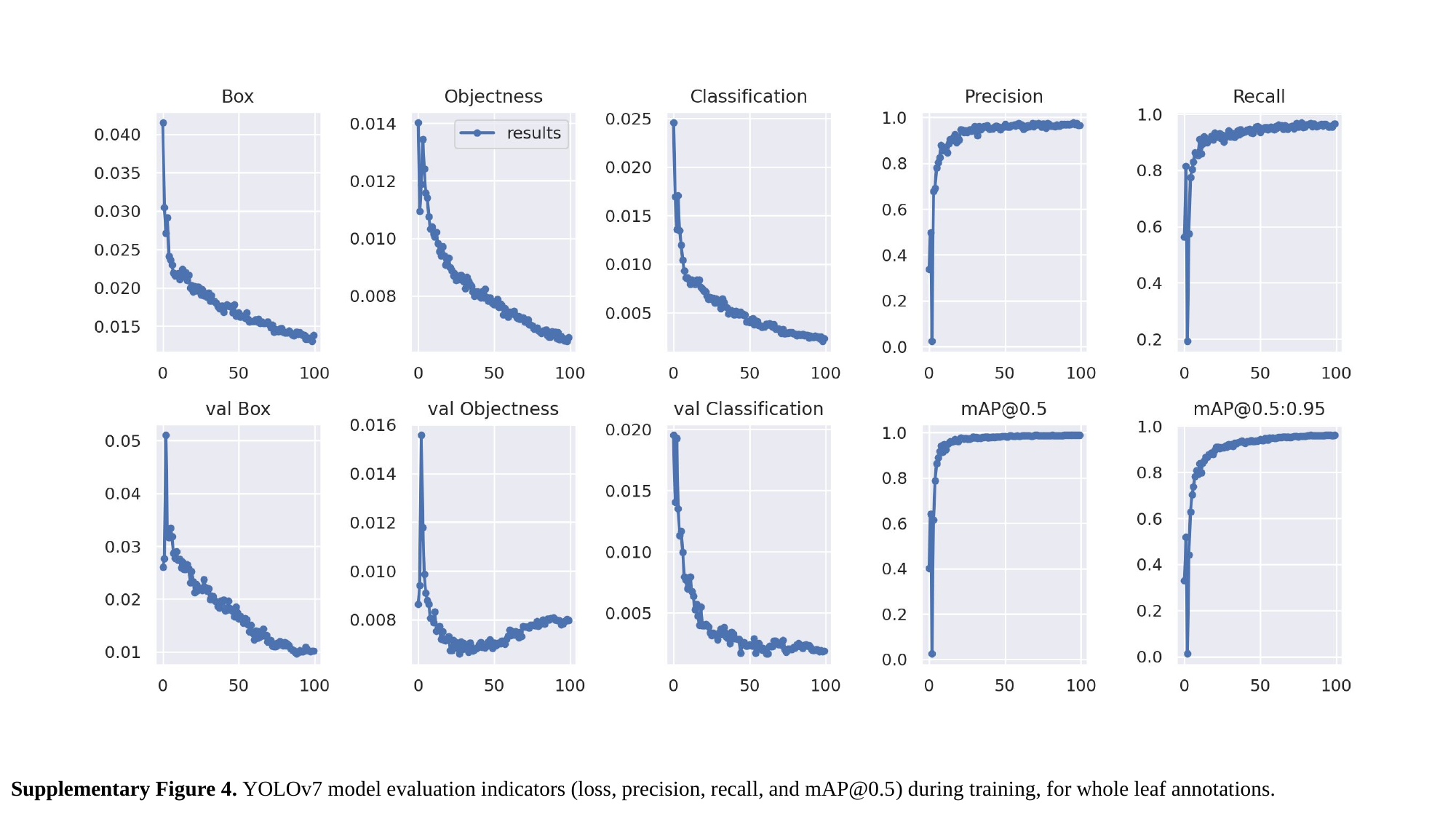

Supplementary Figure 4. YOLOv7 model evaluation indicators (loss, precision, recall, and mAP@0.5) during training, for whole leaf annotations.

## Slide 5
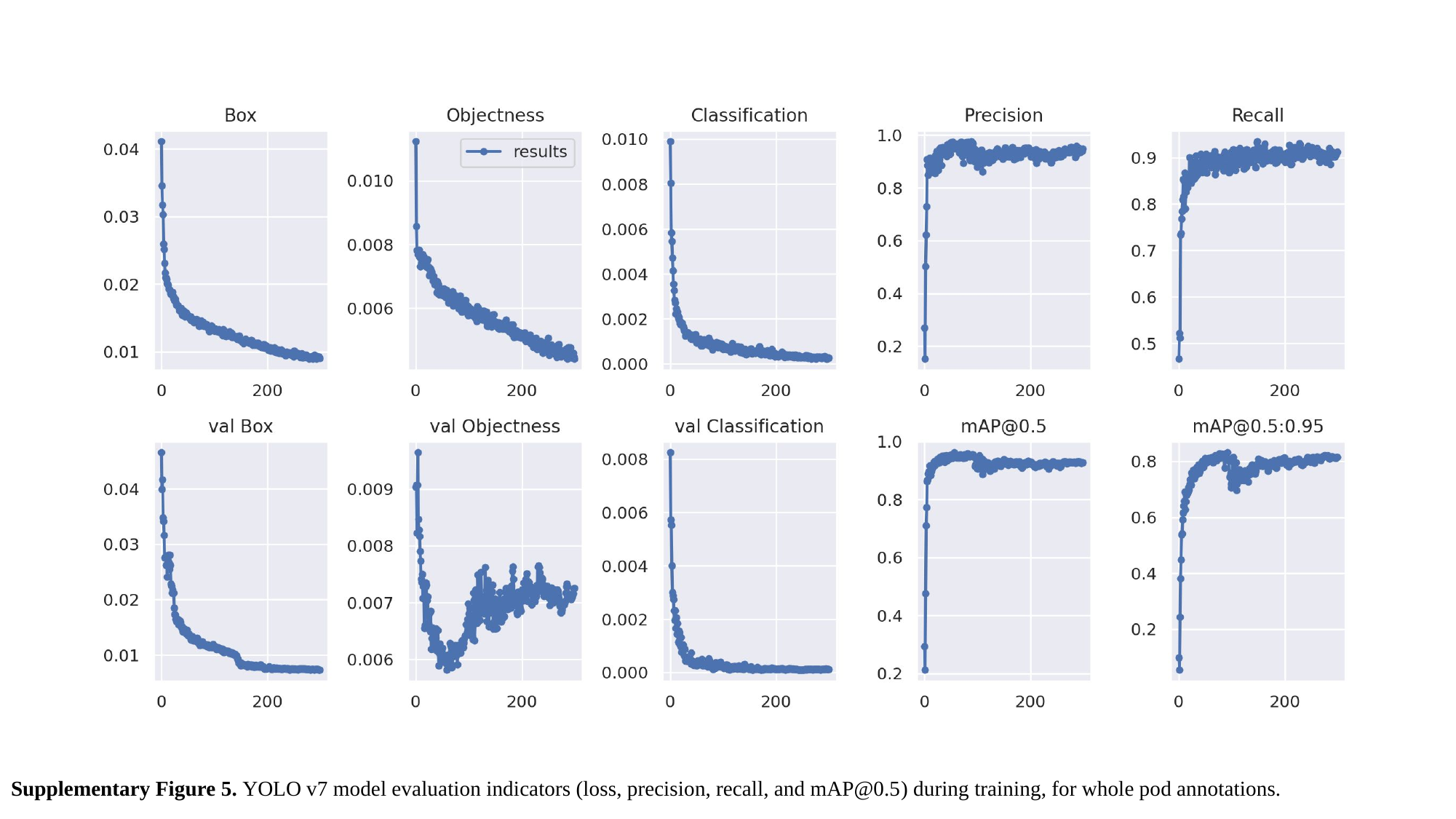

Supplementary Figure 5. YOLO v7 model evaluation indicators (loss, precision, recall, and mAP@0.5) during training, for whole pod annotations.

## Slide 6
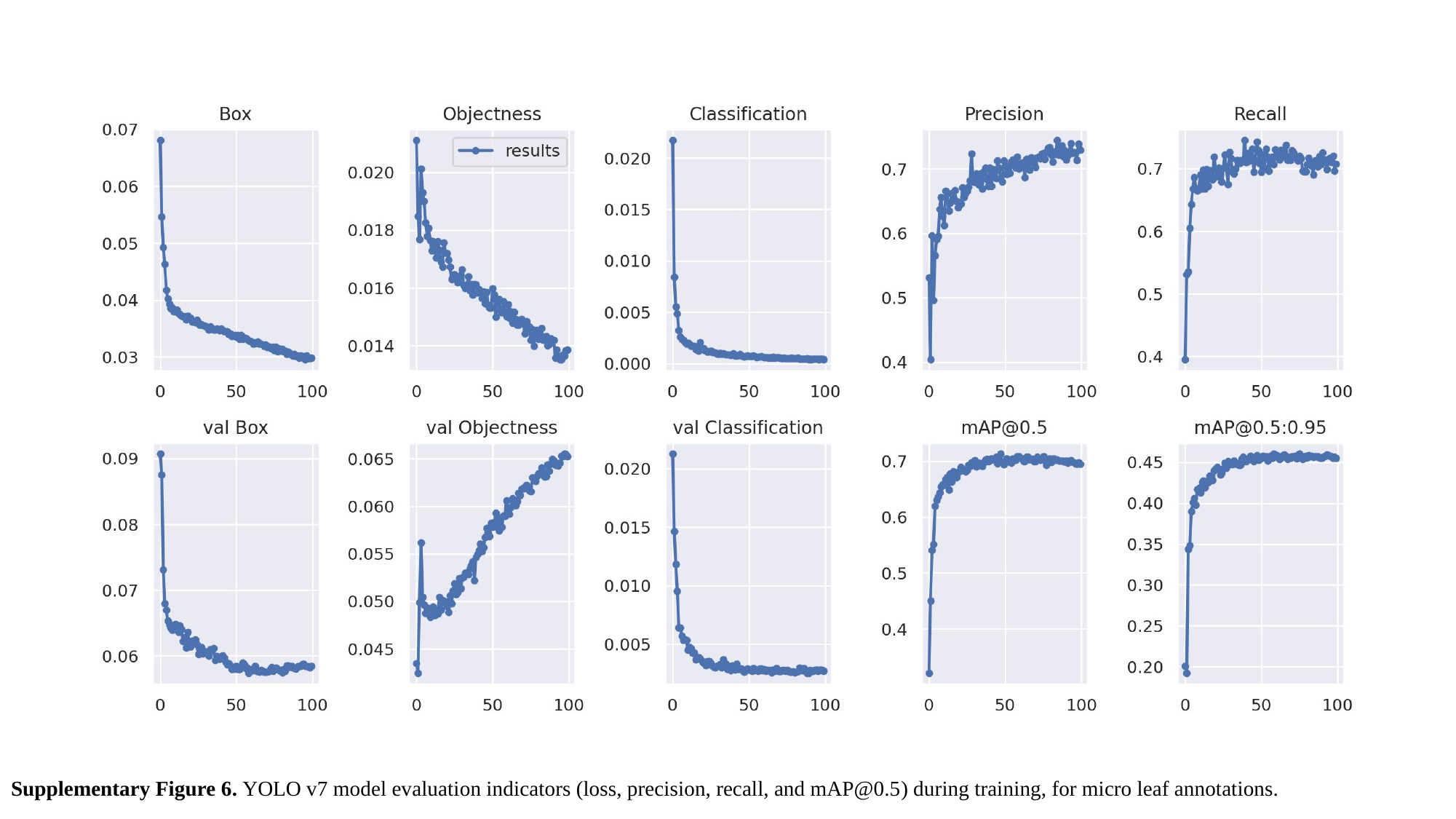

Supplementary Figure 6. YOLO v7 model evaluation indicators (loss, precision, recall, and mAP@0.5) during training, for micro leaf annotations.

## Slide 7
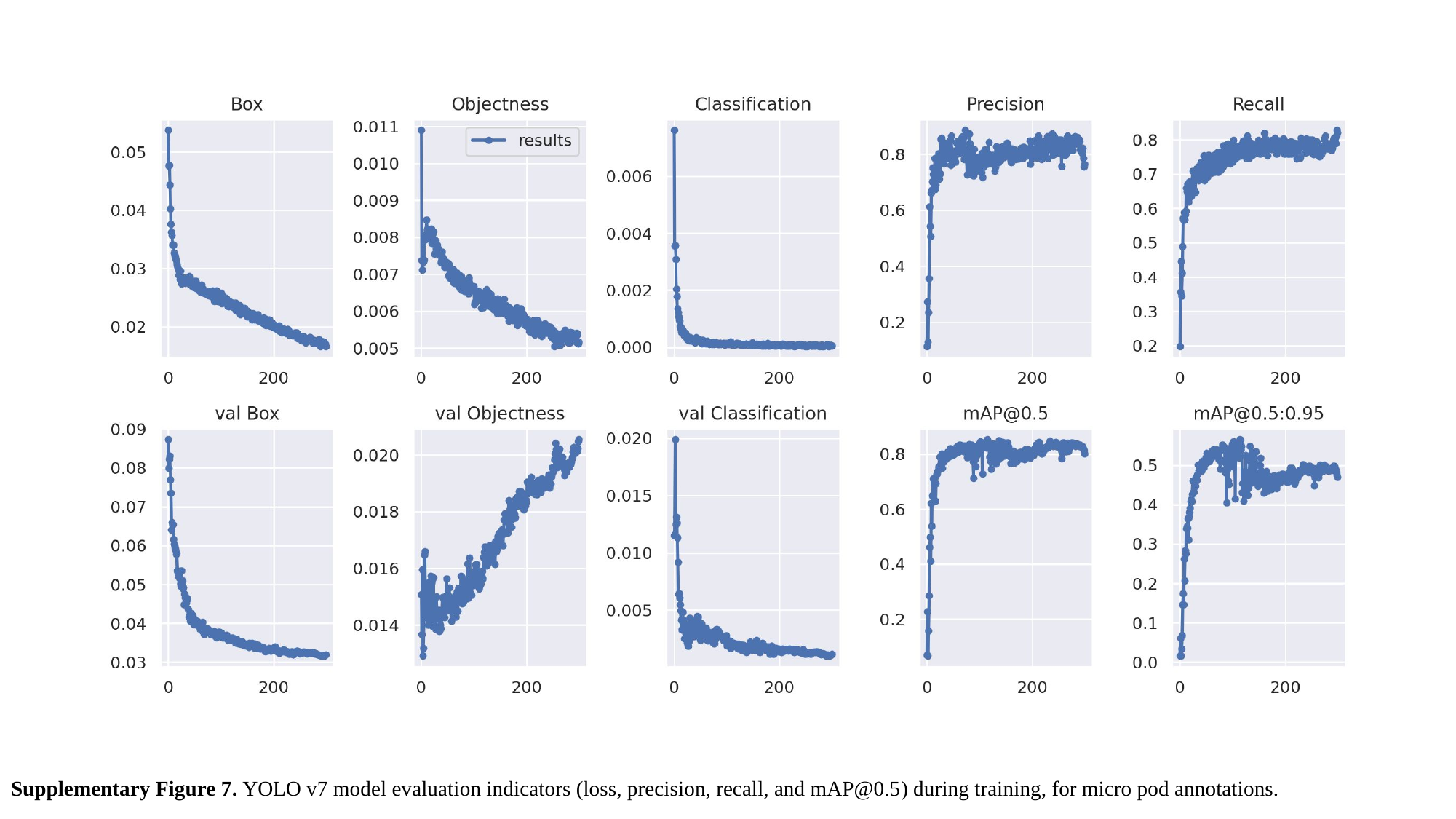

Supplementary Figure 7. YOLO v7 model evaluation indicators (loss, precision, recall, and mAP@0.5) during training, for micro pod annotations.

## Slide 8
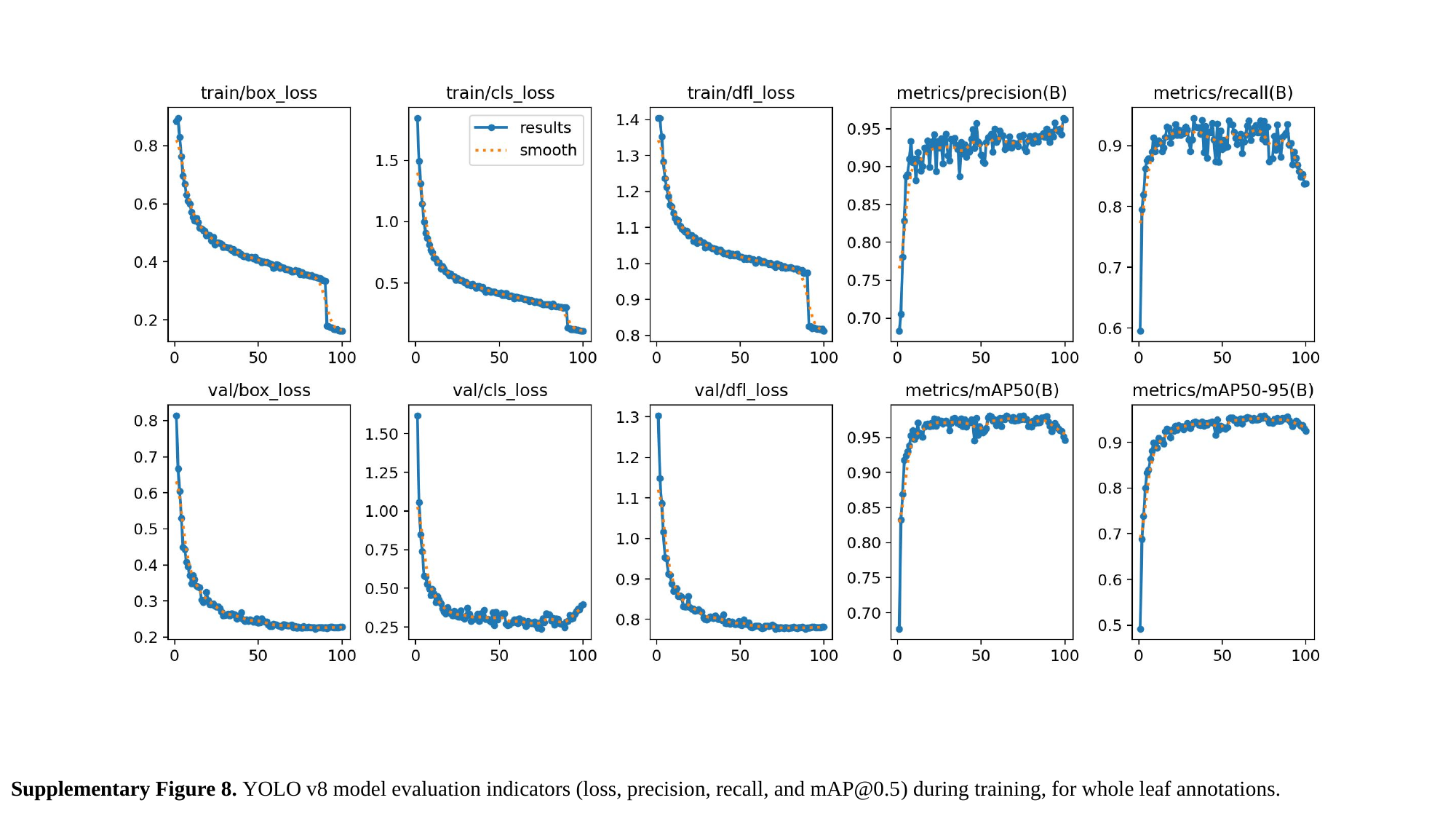

Supplementary Figure 8. YOLO v8 model evaluation indicators (loss, precision, recall, and mAP@0.5) during training, for whole leaf annotations.

## Slide 9
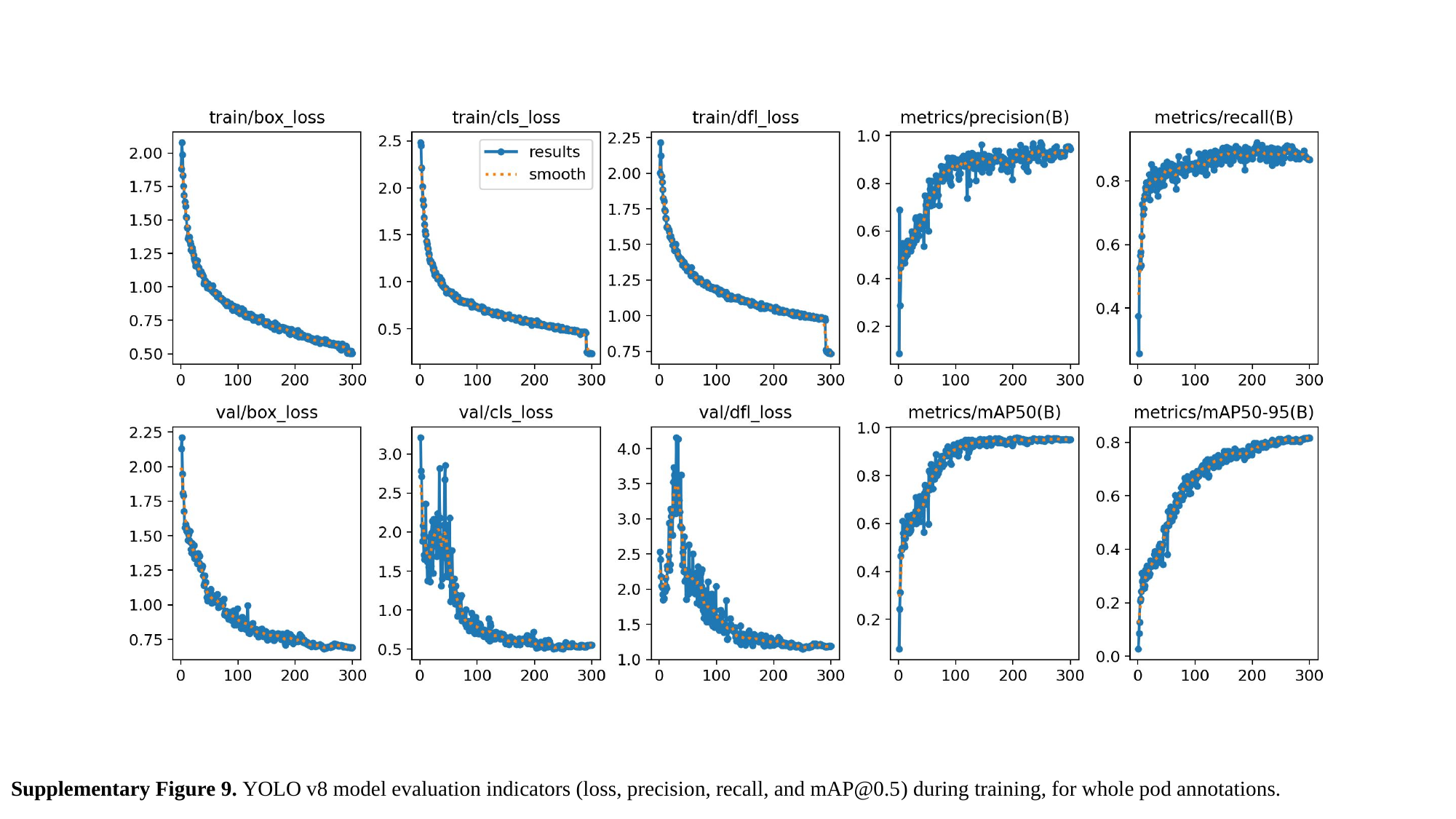

Supplementary Figure 9. YOLO v8 model evaluation indicators (loss, precision, recall, and mAP@0.5) during training, for whole pod annotations.

## Slide 10
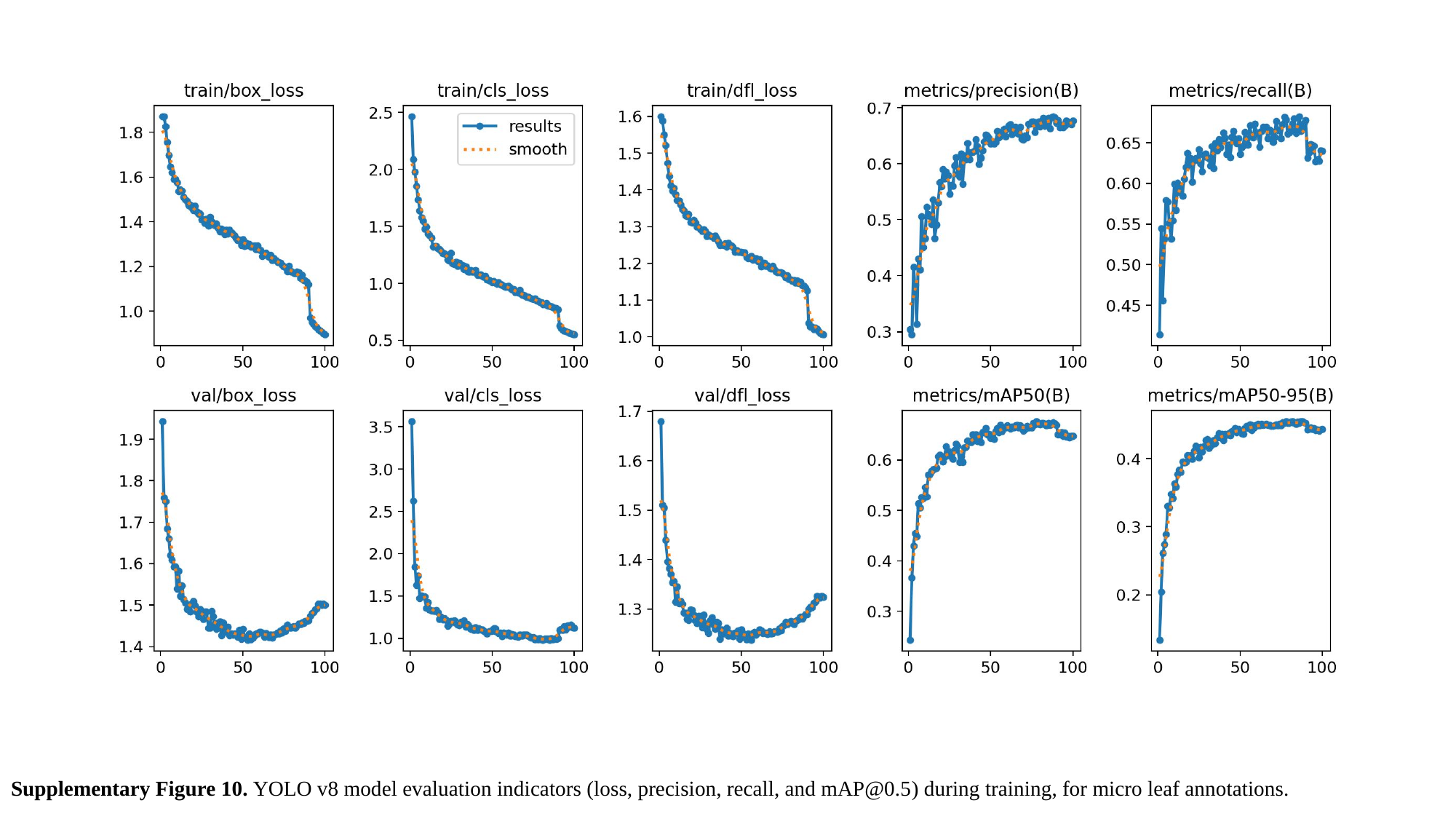

Supplementary Figure 10. YOLO v8 model evaluation indicators (loss, precision, recall, and mAP@0.5) during training, for micro leaf annotations.

## Slide 11
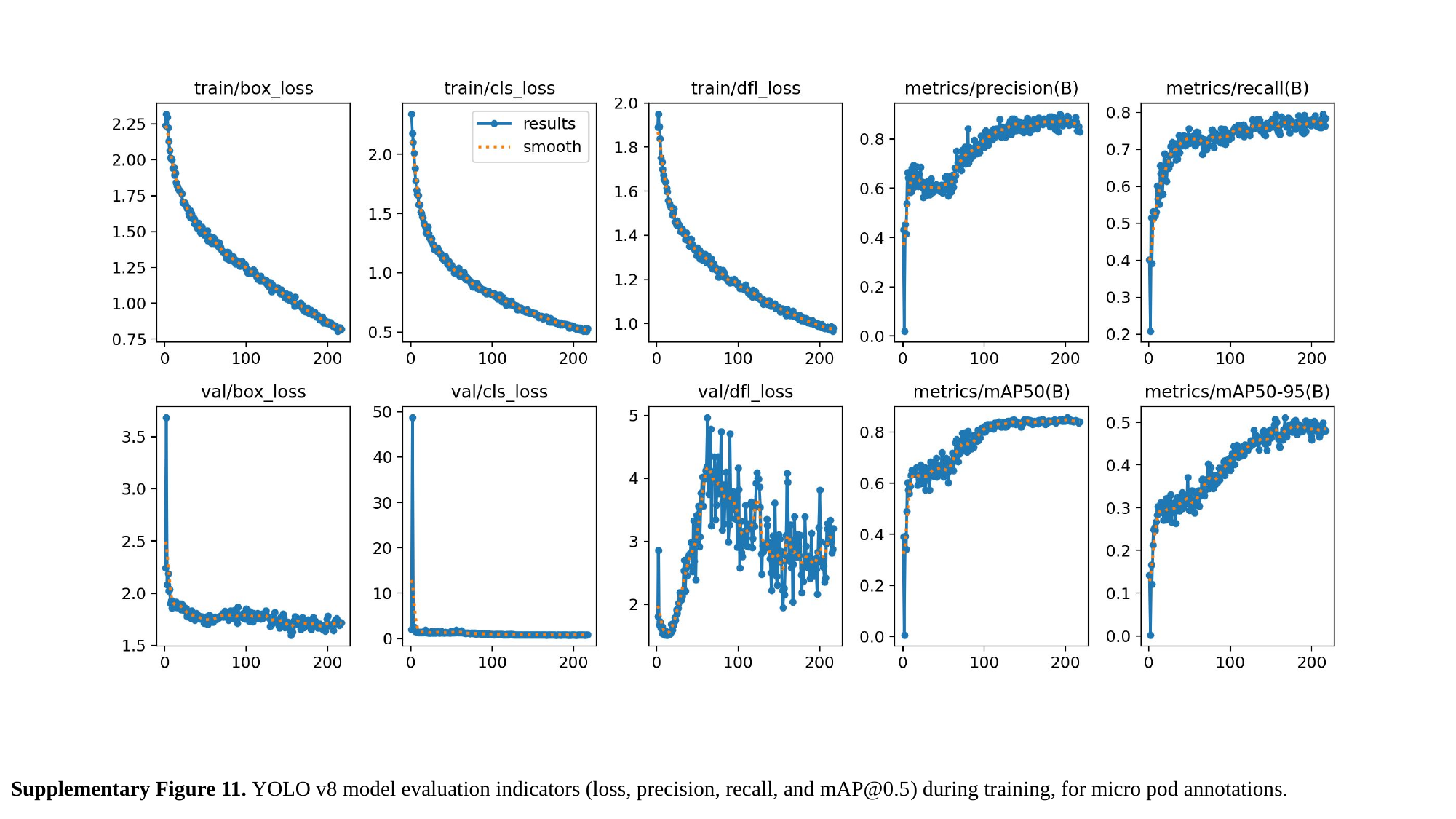

Supplementary Figure 11. YOLO v8 model evaluation indicators (loss, precision, recall, and mAP@0.5) during training, for micro pod annotations.

## Slide 12
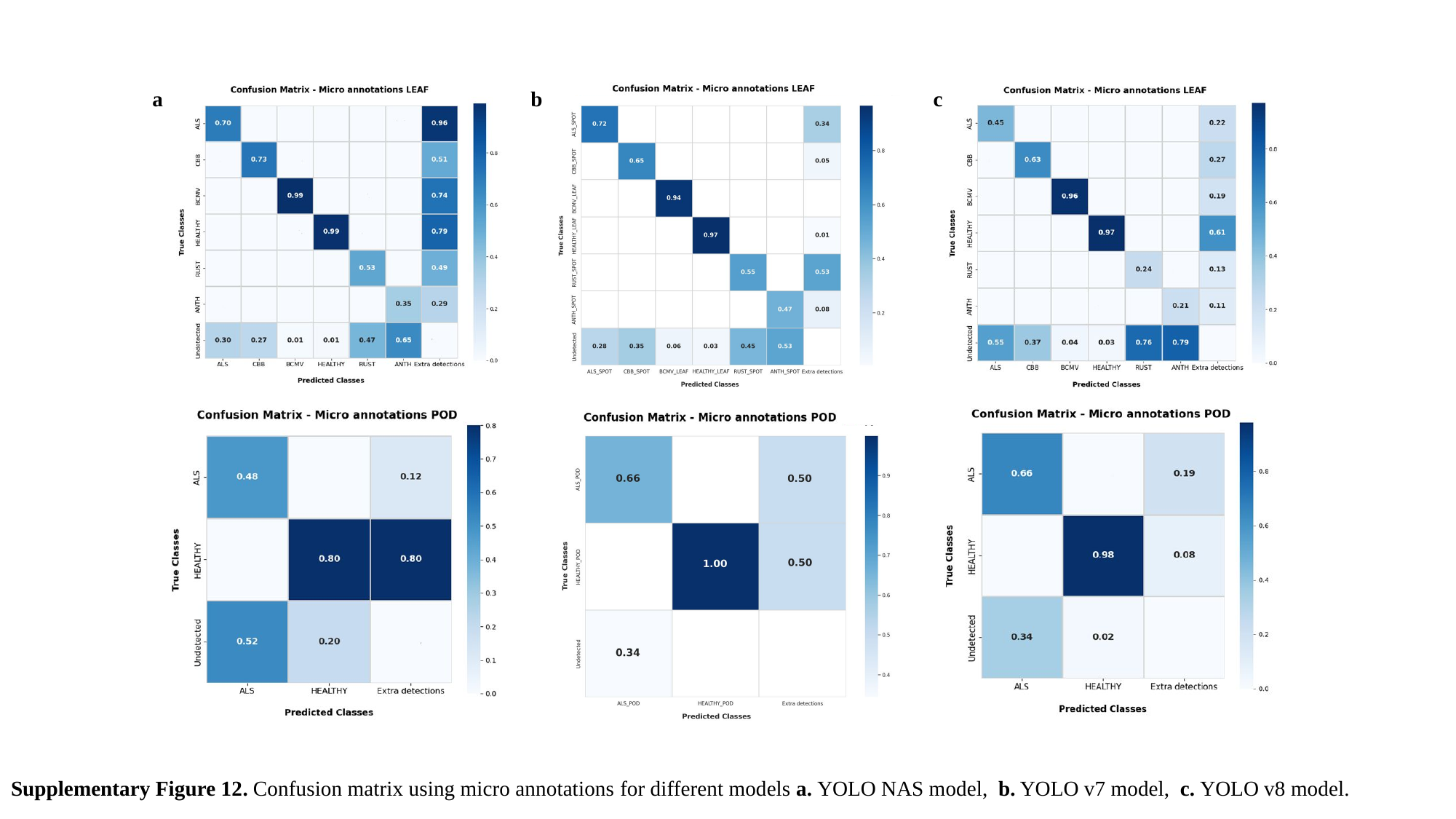

a
b
c
Supplementary Figure 12. Confusion matrix using micro annotations for different models a. YOLO NAS model,  b. YOLO v7 model,  c. YOLO v8 model.

## Slide 13
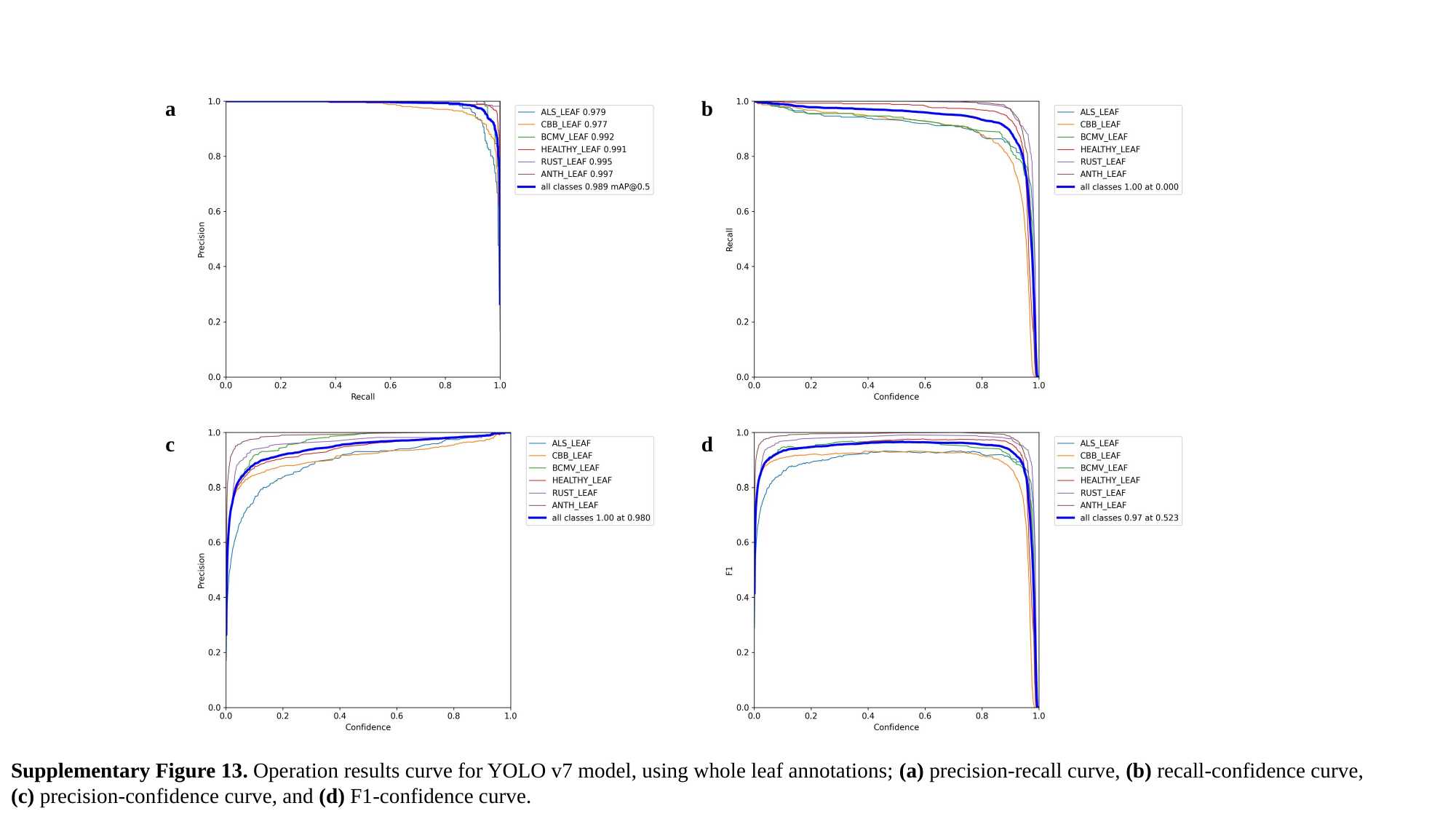

b
a
c
d
Supplementary Figure 13. Operation results curve for YOLO v7 model, using whole leaf annotations; (a) precision-recall curve, (b) recall-confidence curve, (c) precision-confidence curve, and (d) F1-confidence curve.

## Slide 14
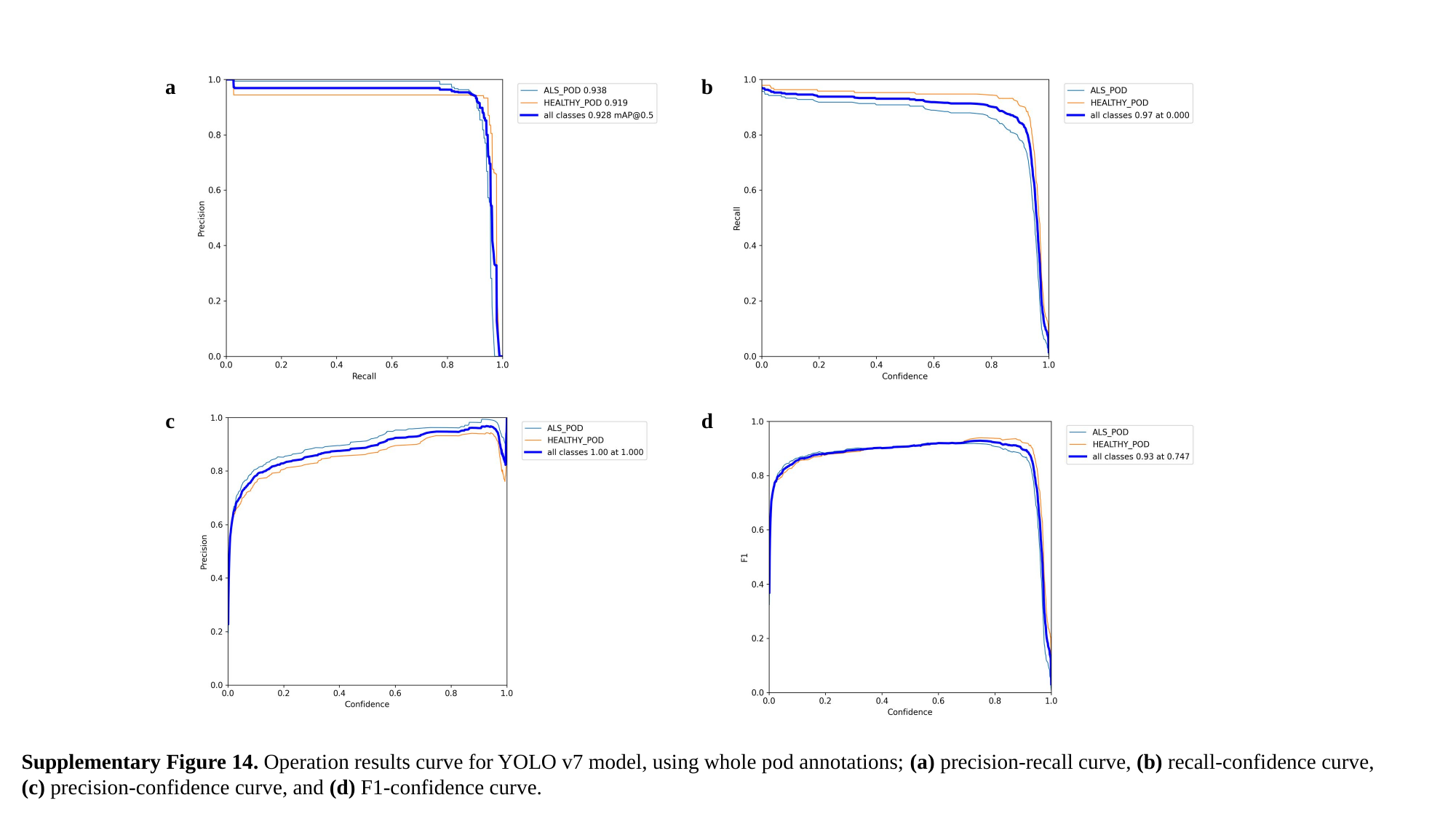

b
a
c
d
Supplementary Figure 14. Operation results curve for YOLO v7 model, using whole pod annotations; (a) precision-recall curve, (b) recall-confidence curve,
(c) precision-confidence curve, and (d) F1-confidence curve.

## Slide 15
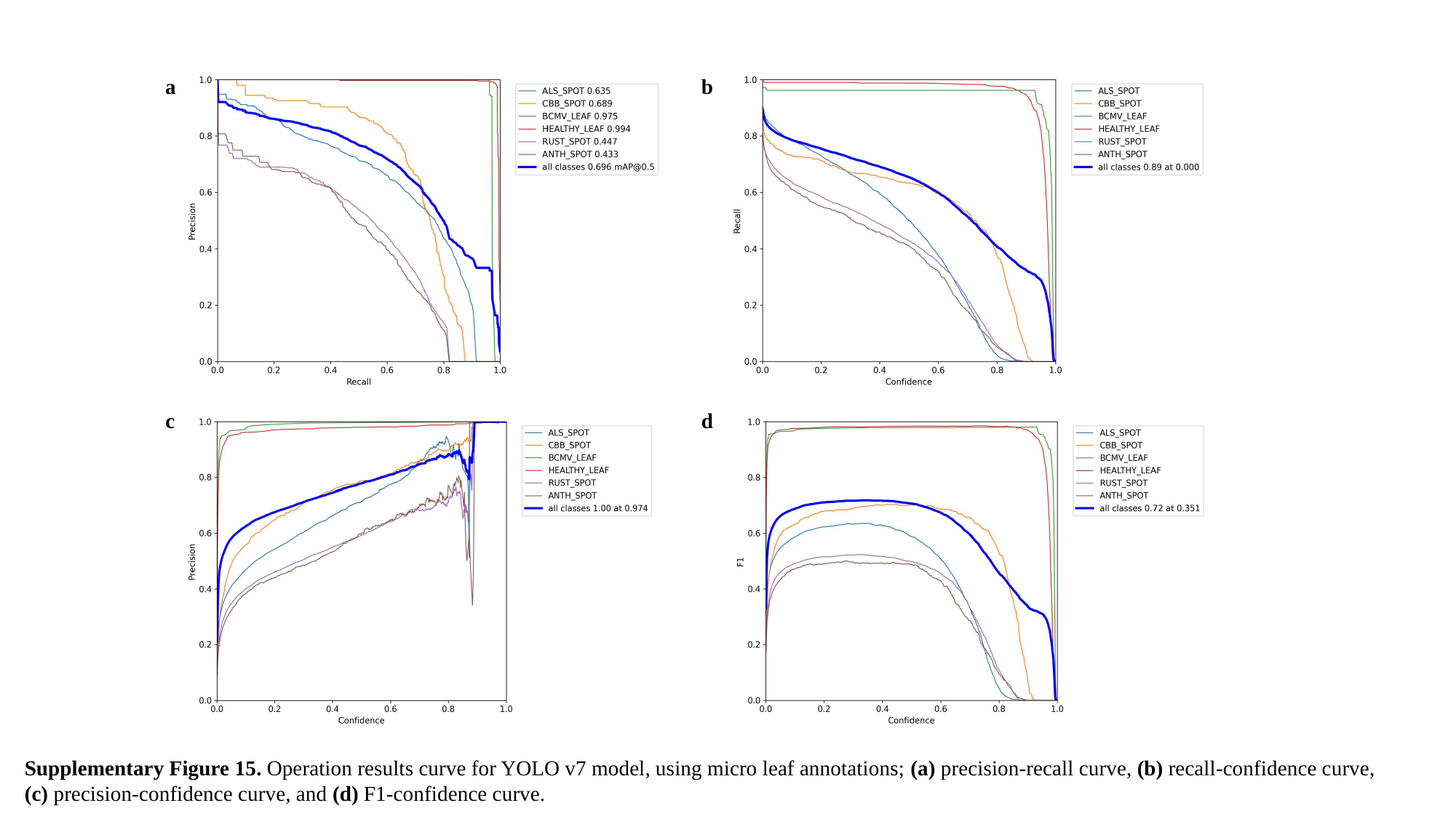

b
a
c
d
Supplementary Figure 15. Operation results curve for YOLO v7 model, using micro leaf annotations; (a) precision-recall curve, (b) recall-confidence curve,
(c) precision-confidence curve, and (d) F1-confidence curve.

## Slide 16
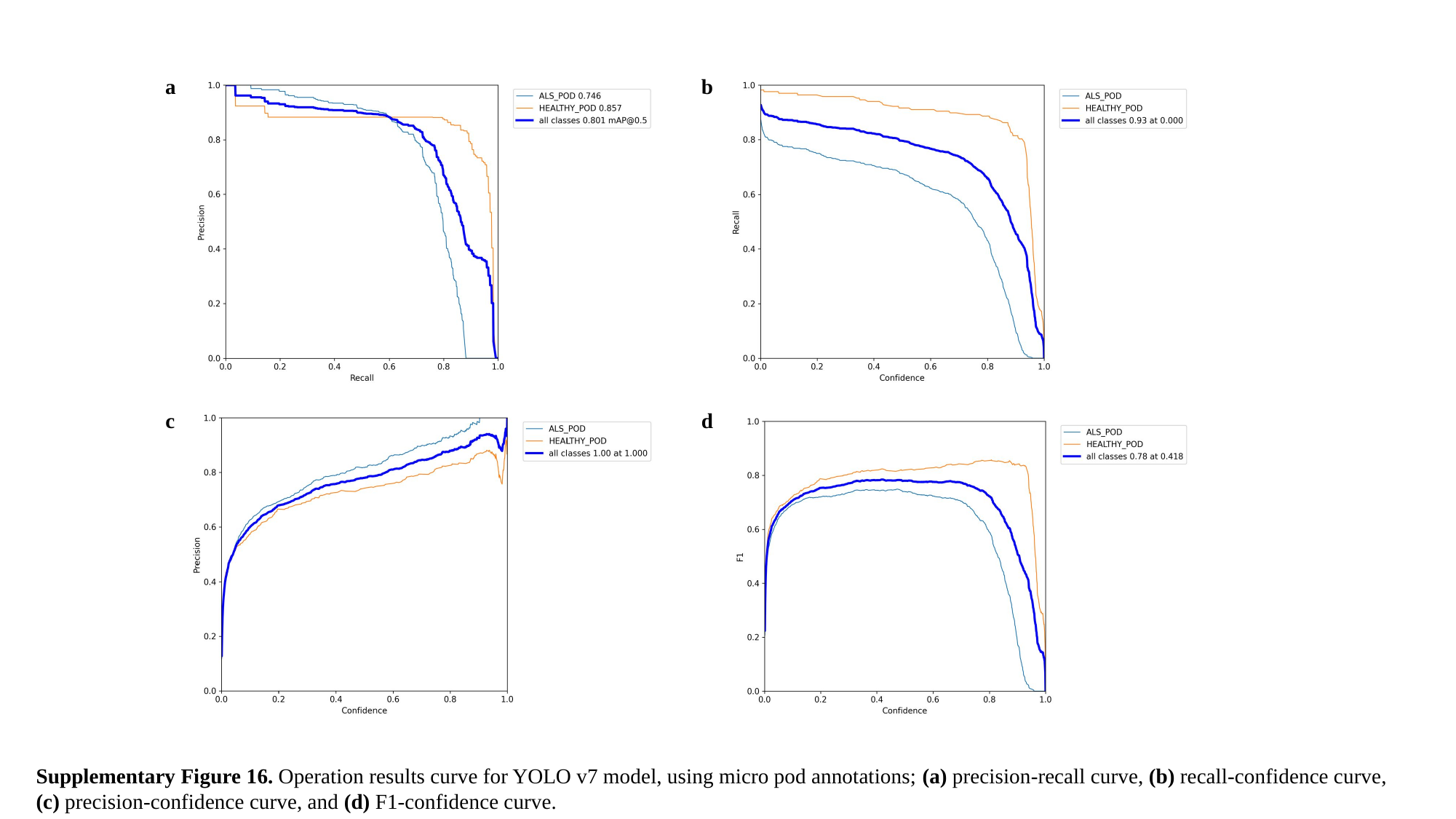

b
a
c
d
Supplementary Figure 16. Operation results curve for YOLO v7 model, using micro pod annotations; (a) precision-recall curve, (b) recall-confidence curve,
(c) precision-confidence curve, and (d) F1-confidence curve.

## Slide 17
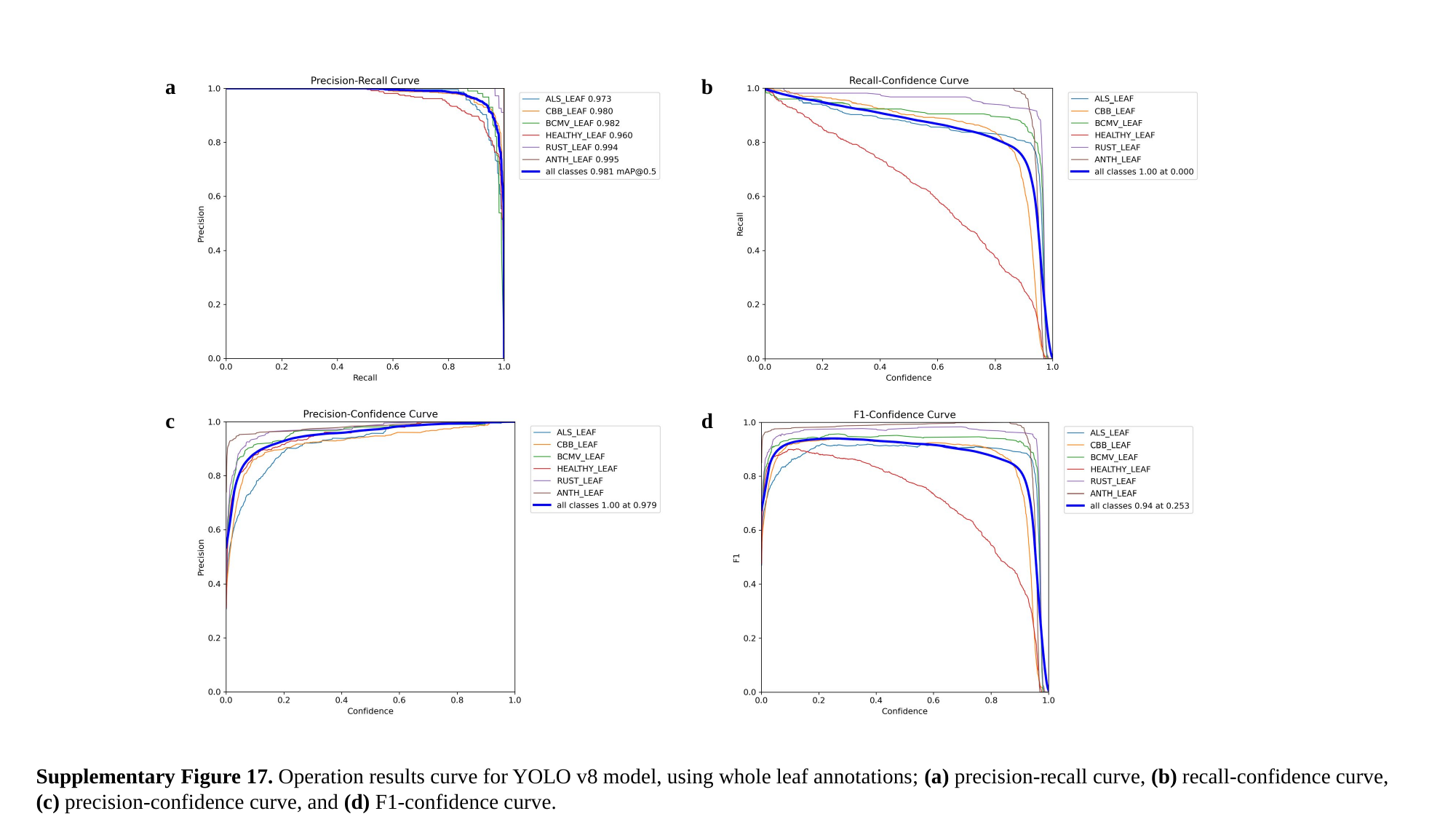

b
a
c
d
Supplementary Figure 17. Operation results curve for YOLO v8 model, using whole leaf annotations; (a) precision-recall curve, (b) recall-confidence curve,
(c) precision-confidence curve, and (d) F1-confidence curve.

## Slide 18
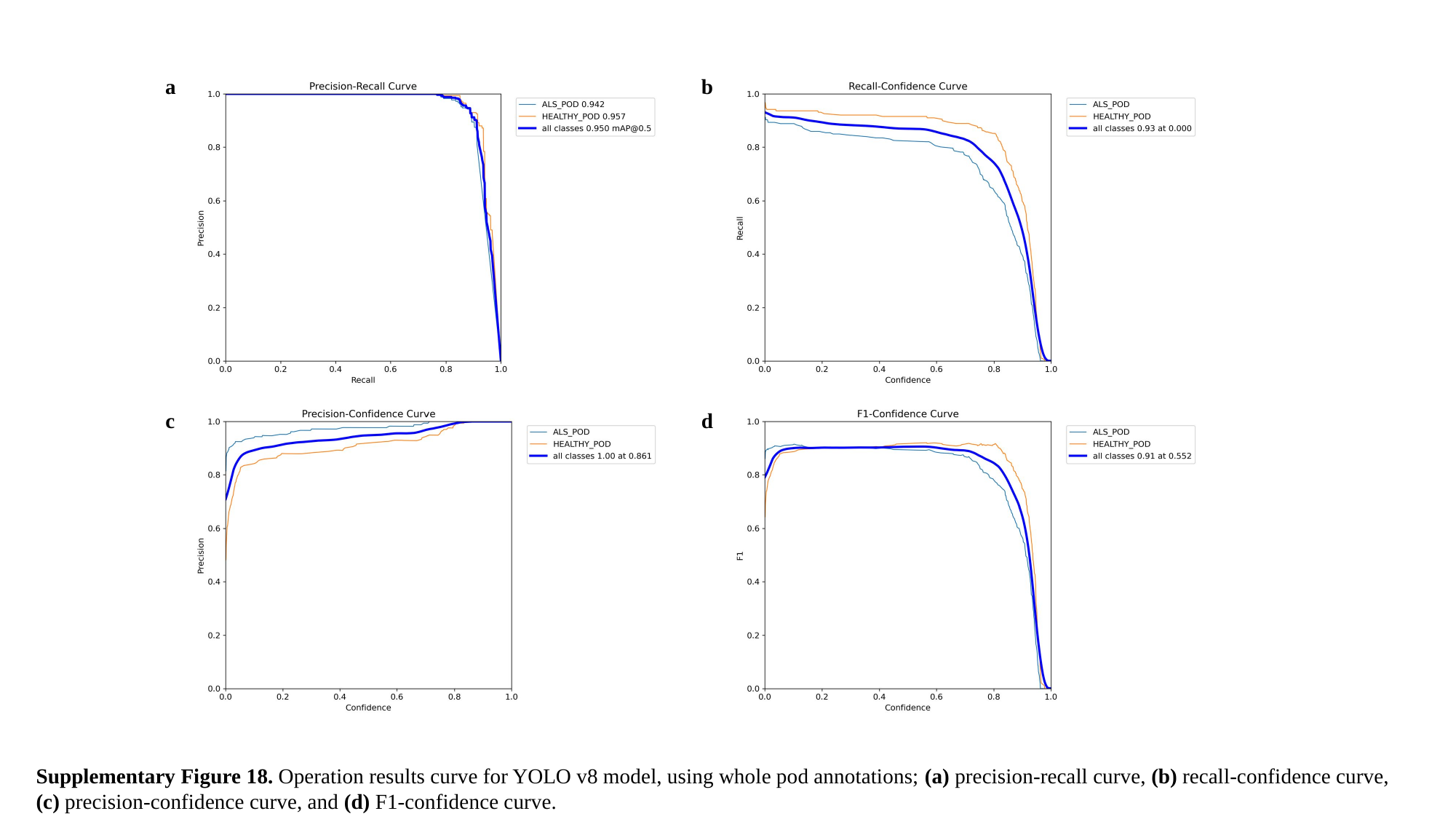

b
a
c
d
Supplementary Figure 18. Operation results curve for YOLO v8 model, using whole pod annotations; (a) precision-recall curve, (b) recall-confidence curve,
(c) precision-confidence curve, and (d) F1-confidence curve.

## Slide 19
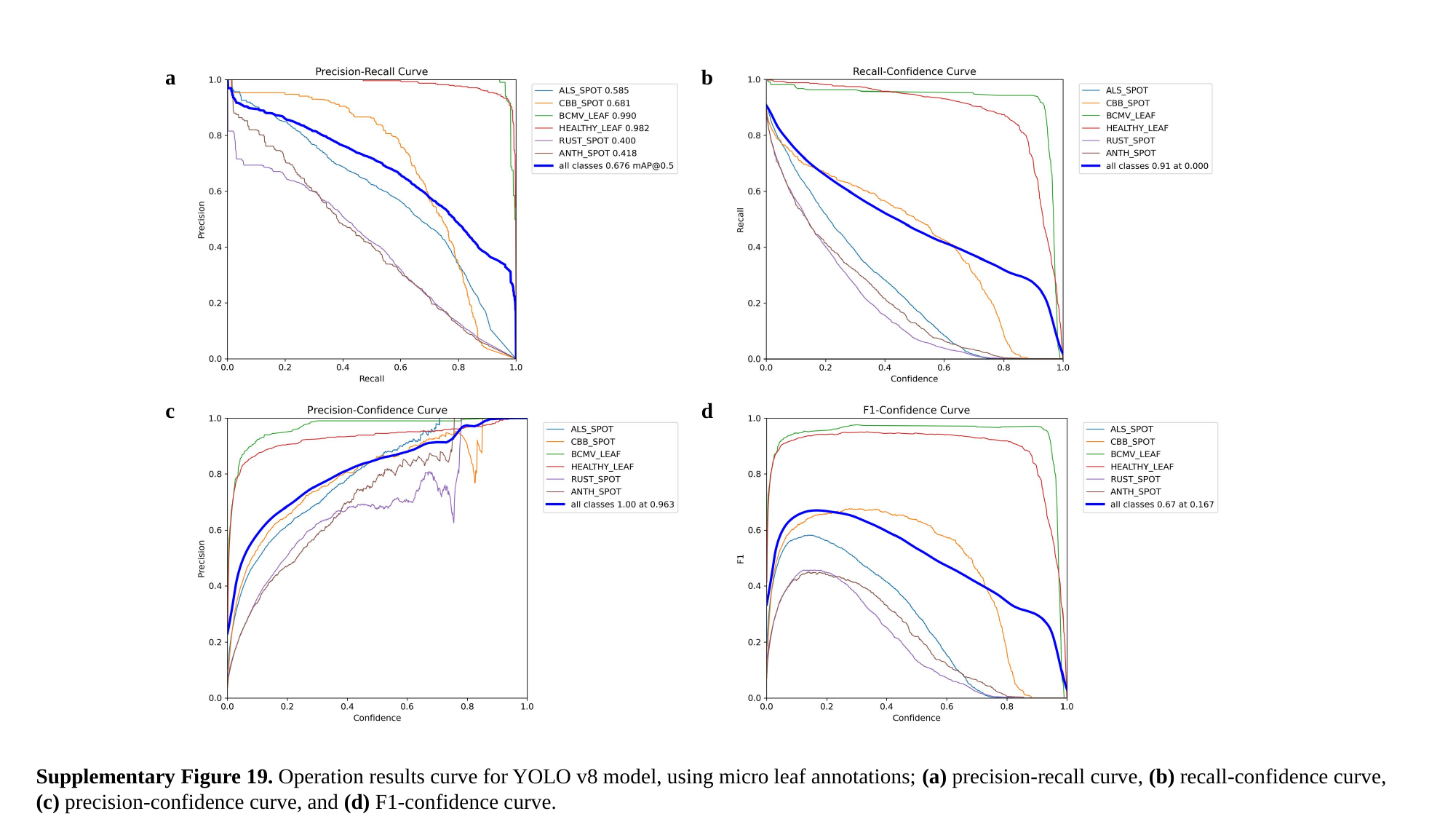

b
a
c
d
Supplementary Figure 19. Operation results curve for YOLO v8 model, using micro leaf annotations; (a) precision-recall curve, (b) recall-confidence curve,
(c) precision-confidence curve, and (d) F1-confidence curve.

## Slide 20
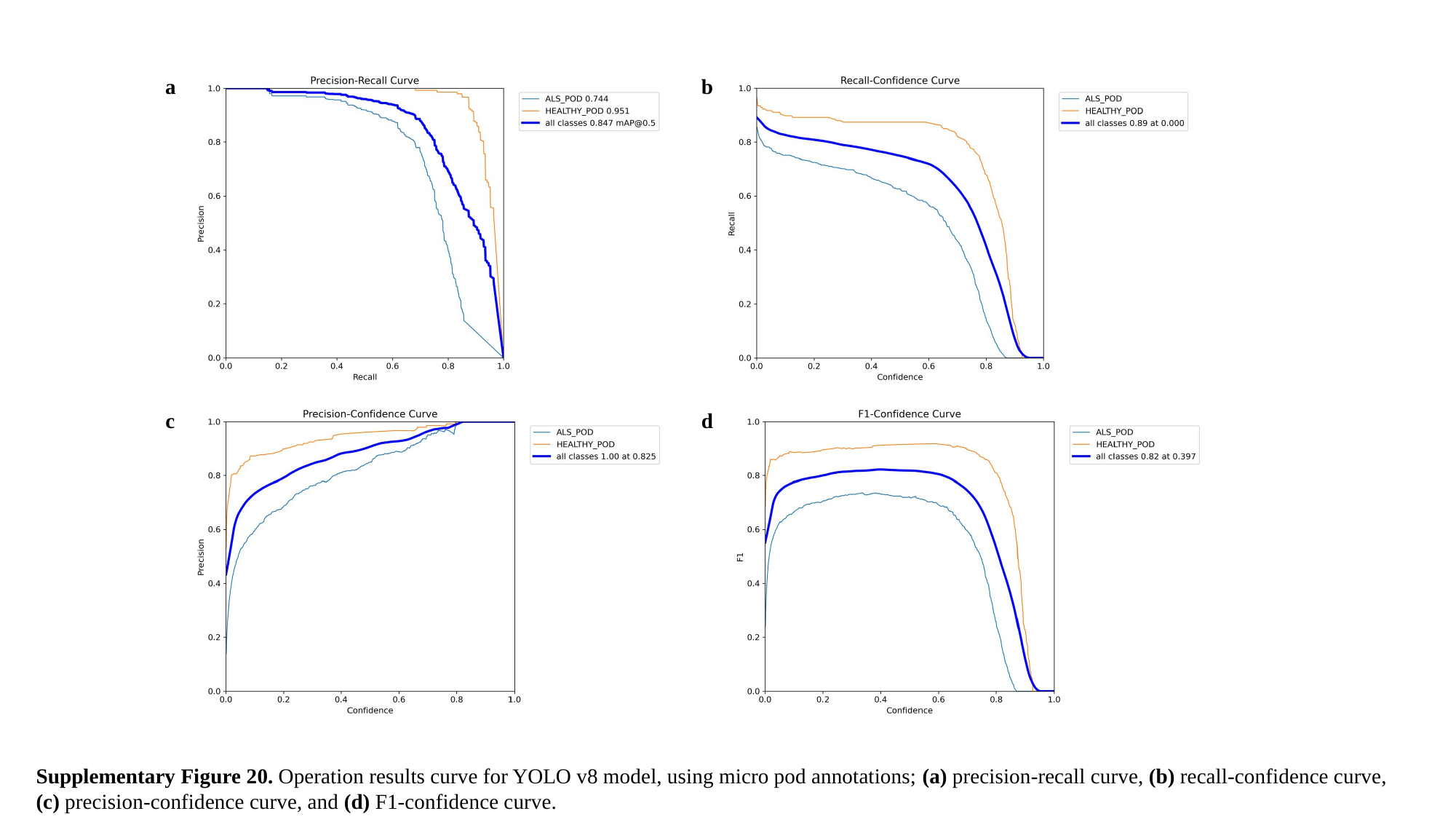

b
a
c
d
Supplementary Figure 20. Operation results curve for YOLO v8 model, using micro pod annotations; (a) precision-recall curve, (b) recall-confidence curve,
(c) precision-confidence curve, and (d) F1-confidence curve.

## Slide 21
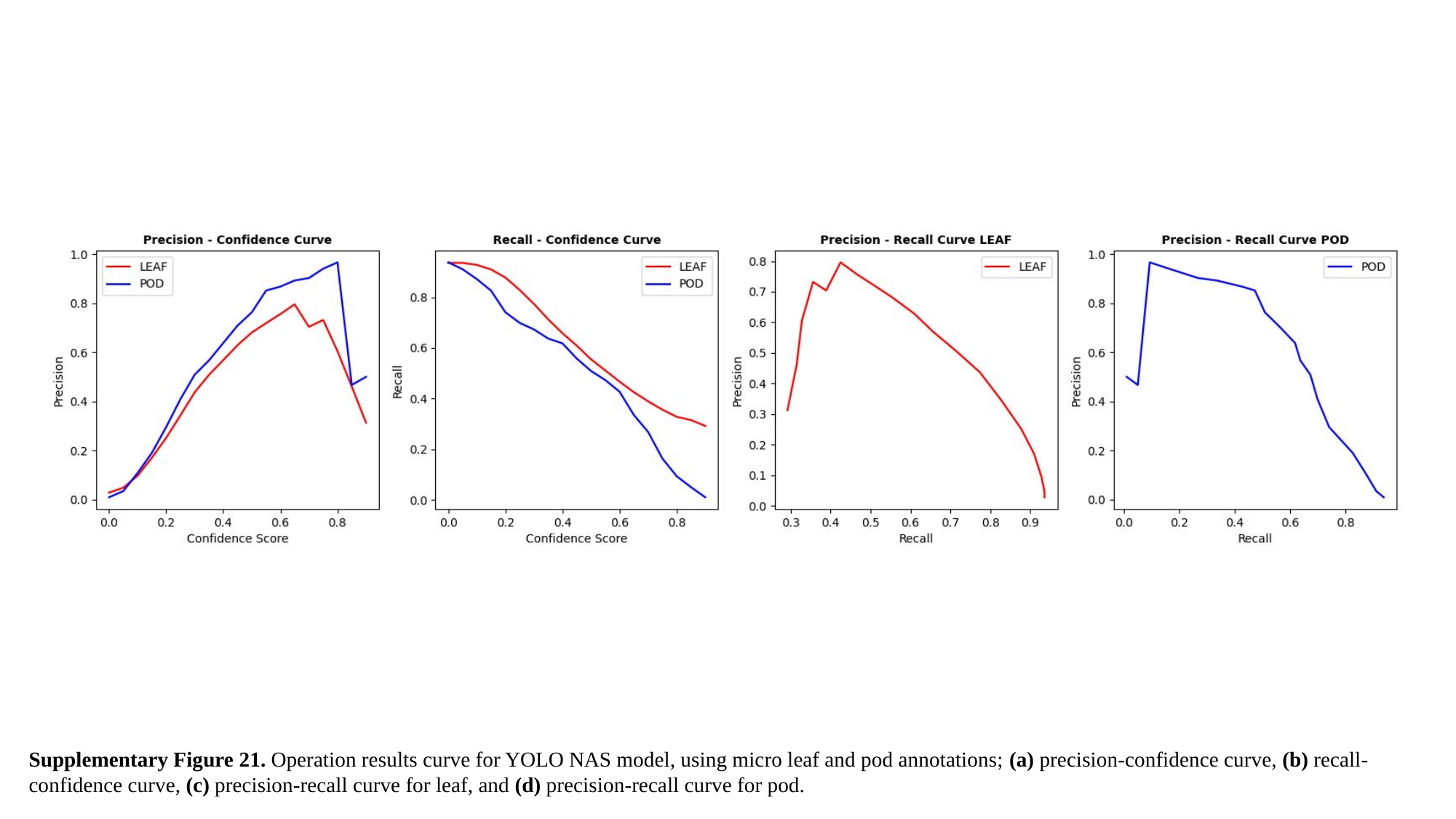

Supplementary Figure 21. Operation results curve for YOLO NAS model, using micro leaf and pod annotations; (a) precision-confidence curve, (b) recall-confidence curve, (c) precision-recall curve for leaf, and (d) precision-recall curve for pod.

## Slide 22
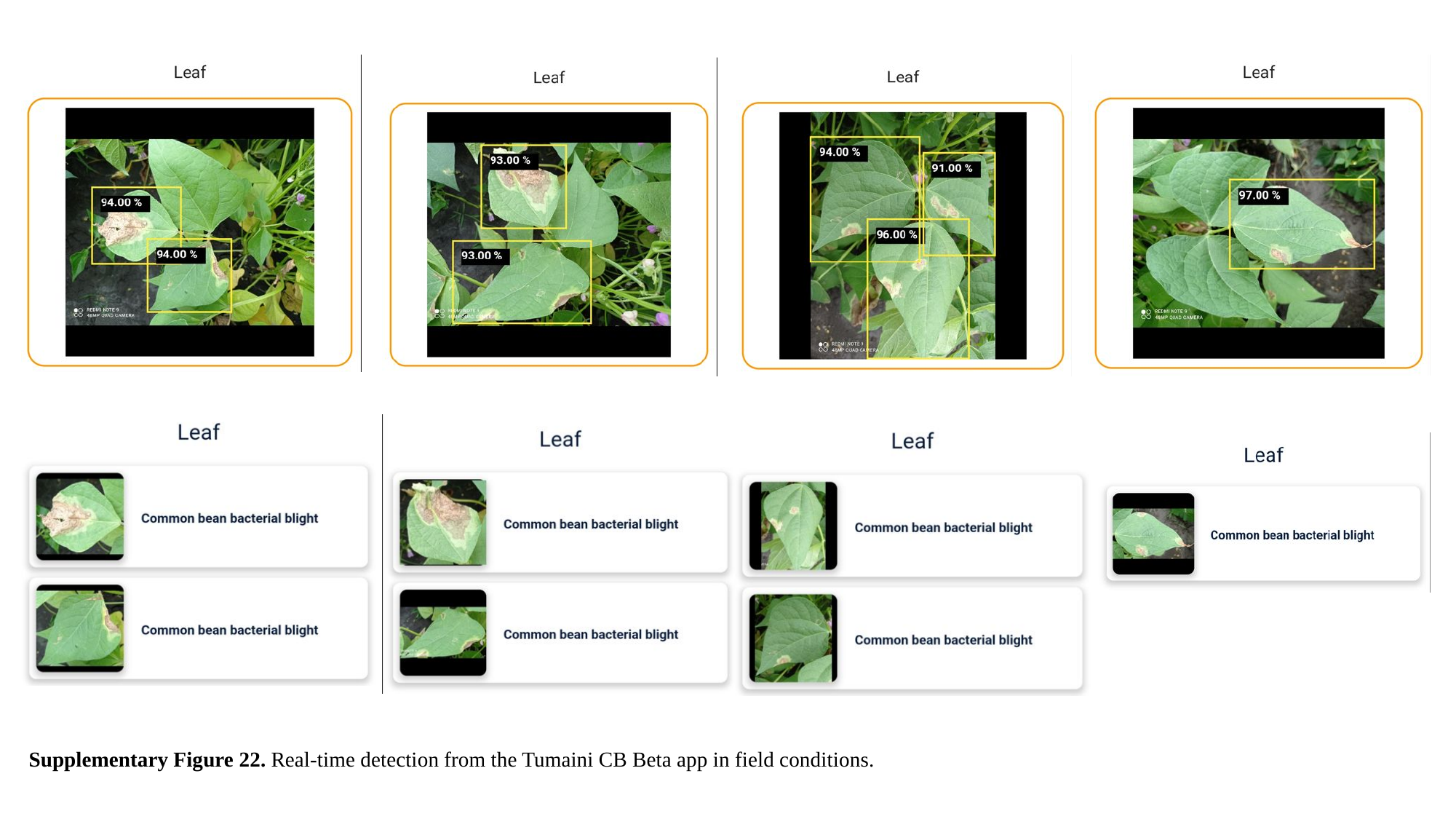

Supplementary Figure 22. Real-time detection from the Tumaini CB Beta app in field conditions.
