# Supplementary material for: Advancing common bean (Phaseolus vulgaris L.) disease detection with YOLO driven deep learning to enhance agricultural AI
Source: Sci Rep. 2024 Jul 6;14:15596. doi: 10.1038/s41598-024-66281-w (PMC11227504; doi:10.1038/s41598-024-66281-w)
Supplement: Supplementary file 2 — Supplementary Tables. [file 41598_2024_66281_MOESM2_ESM.docx]

**Supplementary Table 1.** Overview of banana data set collections, locations, and image acquisition.

| **Country** | **Image type** | **Cell phone used** | **Genotype** |
| --- | --- | --- | --- |
| Colombia | Healthy Leaves  Common Bacterial Blight | Xiaomi Redmi Note 8 | DAB 973  AMADEUS  VAX 3  SER 350  ICA PIJAO |
| Tanzania | Angular Leaf Spot (Leaves)  Angular Leaf Spot (Pods)  Common Bean Mosaic Virus | Samsung Galaxy Note 10 + |  |
| Uganda | Healthy Leaves  Healthy Pods  Common Bean Mosaic Virus  Anthracnose  Angular Leaf Spot (Leaves)  Common Bacterial Blight | Tecno Camon 18 | G5686  VEF 2021-232  VEF 2021-233  SMN13  SMN18  NUS 14 |

**Supplementary Table 2.** Description of major common bean diseases and symptoms with their control measures

| **Disease name** | **Description** | **Symptoms** | **Control and References** |
| --- | --- | --- | --- |
| **Angular leaf spot** (ALS) | **Angular leaf spot** (ALS), caused by the fungus *Phaeoisariopsis* *griseola*, is considered a serious **disease** of **beans** in many regions. It attacks bean stems, leaves and pods. | Spots originating on the lower leaf surface are delimited by the veins and veinlets and develop into grey lesions which later turn light-brown. Lesions may be surrounded by a chlorotic halo, but they lack a coloured border. The striking angularity of the spots is a diagnostic feature of *P. griseola*. The lesions may be so numerous as to cause premature defoliation. Stem lesions caused by *P. griseola* are elongate and brown.  Lesions on pods are less frequent than on leaves. They are oval to circular, superficial at first, with nearly black margins and reddish-brown centres, both of which are sharply defined. The spots vary in size and, ultimately, may become so crowded that they coalesce and occupy the width of the pod. | Follow crop rotation, possibly with 2 years between bean crops to allow for decomposition  of plant residues,   - Use of disease-free and resistant variety seeds - Use appropriate chemicals  [Michelle M. Nay](https://pubmed.ncbi.nlm.nih.gov/?term=Nay%20MM%5BAuthor%5D) *et al*., (2019);Misganaw Aytenfsu *et al*., (2023) |
| Common bean mosaic virus  (CBMV) | CBMV causes common mosaic and necrosis (black root) in Phaseolus vulgaris. The type of symptom produced is determined by the strain of CBMV, temperature and the host genotype. | Symptoms associated with common mosaic include leaf rolling or blistering, light and dark-green patches on the leaf (green mosaic), chlorotic vein banding, yellow mosaic and growth reduction. Mottling and malformation of the primary leaves is an indication that the primary infection occurred through seed. Cultivars which develop common mosaic may have distinct chlorotic or necrotic local lesions. | Possible control measures for BCMV include planting healthy seed, improving cultural practices and using appropriate chemicals.  [Elizabeth A Worrall](https://pubmed.ncbi.nlm.nih.gov/?term=Worrall+EA&cauthor_id=26111585) *et al*., (2015);  Muning Tang and Xue Feng (2023) |
| Common bean bacterial blight (CBB) | Common blight, halo blight, and bacterial brown spot are bacterial leaf spot diseases of beans. Common blight is caused by the bacterium *Xanthomonas axonopodis* pv. *Phaseoli*.The symptoms expressed and the life cycles of these three bacterial diseases are all very similar. Therefore, making an accurate diagnosis can be difficult. Fortunately, all three diseases can be controlled with the same practices. | Most bacterial leaf infections initially appear as a small angular or circular, water-soaked (dark green) spot. As these infections develop, the spots turn brown and dry, while a yellow halo of leaf tissue encircles the brown spot. Severely infected leaves often appear yellow and shredded as the dry, brown spots disintegrate or fall from the leaf. | Use resistant bean varieties whenever possible. Remove and dispose of all plant debris at the end of the growing season. Diseased plants should not be composted since the pile does not reach a high enough temperature over winter to kill bacterial organisms. Practice crop rotation and avoid planting beans in the same location for more than three years in a row. To minimize the spread of disease, avoid overhead irrigation and stay out of the garden when plants are wet. Remove all infected portions of the plant or the entire plant from the garden, as soon as disease is detected.  [Nicolas W. G. Chen](https://bsppjournals.onlinelibrary.wiley.com/authored-by/Chen/Nicolas+W.+G.) *et al.,* (2021) |
| Common bean Anthracnose (CBA) | Anthracnose disease plays major role in reducing common bean grain yield in Africa. It is caused by seed-borne fungal pathogen *Colletotrichum lindemuthianum*.  Bean anthracnose attacks leaves, stems, pods and seeds, causing dark brown necrotic lesions that decrease leaf photosynthetic activity. Reduced photosynthesis results in leaf senescence, stunted bean growth and eventual death. Yield loss of up to 100% due to anthracnose has been reported in Africa. | During disease development, a brick red to purplish discoloration is observed on the veins on the lower surface of the leaf. Anthracnose disease symptoms extend on the upper surface of the leaf and at the base of the stem, progressing upwards and producing dark brown to black lesions along the veins. Disease symptoms are also observed on bean pods, causing dark red sunken spots and finally on bean seeds. In severe infections, young pods shrivel and dry prematurely. When many pods are infected, the number of seeds infected increases and grain yield and seed quality decreases (Mohammed 2013) | Anthracnose can be managed by crop rotation, planting resistant varieties, foliar application of plant extracts, seed treatment and foliar application of contact or systemic fungicides. Cultivation of resistant varieties is the most effective and efficient method of anthracnose management, because the major transmission and survival structure for the anthracnose pathogen is the seed, in which the pathogen can survive for up to five years. Movement of infected seed between sites increases the chance of spreading anthracnose from one location to another. To avoid this, farmers are advised to use improved bean varieties.  Integration of soil solarization, seed treatment and foliar spray with systemic and contact fungicide effectively reduces anthracnose epidemics.  Ana Carolina da Costa Lara Fioreze *et al.,* (2018)  Edith L. Kadege *et al*., (2022) |
| Common Bean Rust | The common bean rust disease has a worldwide distribution, and it occurs in most dry and snap bean productions areas of the world, and most especially in locations where humid to moderately humid conditions, long dew periods, and cool conditions prevail during the bean growing season. Bean rust rarely occurs under dry conditions in arid climates. | Symptoms of the bean rust disease can occur on most aerial parts of the common bean plant but are most often observed on the leaves. These symptoms also occur on pods and sometimes on branches and stems, albeit rarely. Bean rust symptoms do not occur on flowers. The most commonly observed symptoms are the rust-colored pustules which are the uredinia containing urediniospores, also called uredospores. This is known as the repeating summer cycle.  In the field, different types of uredinial pustules (large and small pustules) have been observed on the same plant, suggesting that different races of this pathogen are present on the same bean plant. Pustules on pods and stems are often elongated. As the summer or growing season advances and the bean plants get older, especially near the end of the growing season, the uredinia can be gradually replaced by the dark telia (dark, almost black pustules) containing the also dark brown to black teliospores. A hypersensitive reaction, typical of some host-pathogen genotype reactions is often observed under greenhouse conditions. This type of reaction is also observed in the field. In some cases, this necrotic lesion can contain a sporulating pustule (Harter & Zaumeyer 1941). | Although several strategies exist for the management of the common bean rust disease, rust management (control) under field conditions in most bean producing countries of the world is accomplished using disease resistant cultivars and fungicides. Several fungicides are available for the effective management of bean rust; however, use of fungicides invariably increase production costs. Fungicides are used mostly for the control of rust in snap beans. As indicated earlier, snap beans in many parts of the world (e.g., Eastern and Southern African countries) are notoriously susceptible to rust. Fungicides for bean rust management are most effective when used in the very early stages of the epidemic and preventatively. Effective fungicides include protectants such as chlorothalonil and dithiocarbamates, and systemic chemicals such as triazoles and carboxins (Liebenberg & Pretorius, 2010). Because the bean rust pathogen is not transmitted with the seed, seed treatments for bean rust control are not usually used or needed. Other effective control measures include crop rotation, removal of volunteer plants, deep plowing to remove bean debris from the soil surface and encourage rotting and avoidance by choice of planting time. |
